# Supplementary figures and images for: Cross-sectional study to assess depression among healthcare workers in Lusaka, Zambia during the COVID-19 pandemic
Source: BMJ Open. 2023 Apr 5;13(4):e069257. doi: 10.1136/bmjopen-2022-069257 (PMC10083529; doi:10.1136/bmjopen-2022-069257)

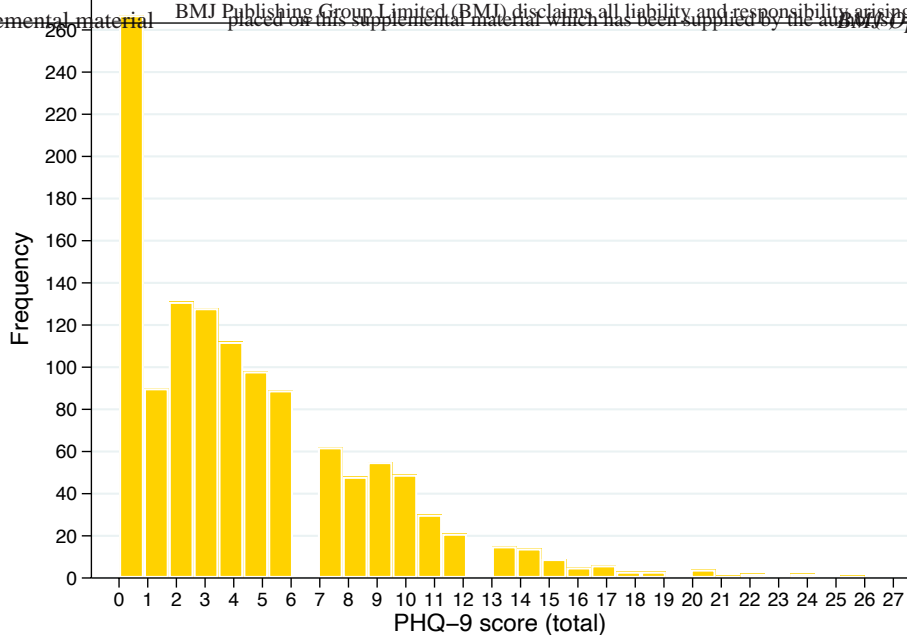

Supplement: Supplementary data [file bmjopen-2022-069257supp001.pdf]
