# Supplementary material for: Cross-sectional study to assess depression among healthcare workers in Lusaka, Zambia during the COVID-19 pandemic
Source: BMJ Open. 2023 Apr 5;13(4):e069257. doi: 10.1136/bmjopen-2022-069257 (PMC10083529; doi:10.1136/bmjopen-2022-069257)
Supplement: Author's manuscript [file bmjopen-2022-069257.draft_revisions.pdf]

BMJ Open is committed to open peer review. As part of this commitment we make the peer review history of every article we publish publicly available.

When an article is published we post the peer reviewers' comments and the authors' responses online. We also post the versions of the paper that were used during peer review. These are the versions that the peer review comments apply to.

The versions of the paper that follow are the versions that were submitted during the peer review process. They are not the versions of record or the final published versions. They should not be cited or distributed as the published version of this manuscript.

BMJ Open is an open access journal and the full, final, typeset and author-corrected version of record of the manuscript is available on our site with no access controls, subscription charges or pay-per-view fees (http://bmjopen.bmj.com).

If you have any questions on BMJ Open's open peer review process please email info.bmjopen@bmj.com

Mental Wellbeing of Health Care Workers in Lusaka, Zambia During the COVID-19 Pandemic

Journal:	*BMJ Open*
Manuscript ID	bmjopen-2022-069257
Article Type:	Original research
Date Submitted by the Author:	17-Oct-2022
Complete List of Authors:	Simbeza, Sandra; Center for Infectious Disease Research in Zambia<br>Mutale, Jacob; Center for Infectious Disease Research in Zambia, Implementation Science Unit<br>Mulabe, Musunge; Center for Infectious Disease Research in Zambia<br>Jere, Lazarus; Center for Infectious Disease Research in Zambia<br>Bukankala, Chama; Center for Infectious Disease Research in Zambia<br>Sikazwe, Izukanji; Center for Infectious Disease Research in Zambia, Chief Executive Officer<br>Bolton-Moore, Carolyn; Center for Infectious Disease Research in Zambia; The University of Alabama at Birmingham School of Medicine, Infectious Disease<br>Mody, Aaloke; Washington University in St Louis, Infectious Diseases<br>Geng, Elvin; Washington University in St Louis School of Medicine<br>Sharma, Anjali; Center for Infectious Disease Research in Zambia<br>Beres, Laura K ; Johns Hopkins University Bloomberg School of Public Health, Department of International Health<br>Pry, Jake; Center for Infectious Disease Research in Zambia, Implementation Science Unit; University of California Davis School of Medicine, Public Health Sciences
Keywords:	COVID-19, EPIDEMIOLOGY, HIV & AIDS < INFECTIOUS DISEASES, MENTAL HEALTH

I, the Submitting Author has the right to grant and does grant on behalf of all authors of the Work (as defined in the below author licence), an exclusive licence and/or a non-exclusive licence for contributions from authors who are: i) UK Crown employees; ii) where BMJ has agreed a CC-BY licence shall apply, and/or iii) in accordance with the terms applicable for US Federal Government officers or employees acting as part of their official duties; on a worldwide, perpetual, irrevocable, royalty-free basis to BMJ Publishing Group Ltd ("BMJ") its licensees and where the relevant Journal is co-owned by BMJ to the co-owners of the Journal, to publish the Work in this journal and any other BMJ products and to exploit all rights, as set out in our licence.

The Submitting Author accepts and understands that any supply made under these terms is made by BMJ to the Submitting Author unless you are acting as an employee on behalf of your employer or a postgraduate student of an affiliated institution which is paying any applicable article publishing charge ("APC") for Open Access articles. Where the Submitting Author wishes to make the Work available on an Open Access basis (and intends to pay the relevant APC), the terms of reuse of such Open Access shall be governed by a Creative Commons licence – details of these licences and which Creative Commons licence will apply to this Work are set out in our licence referred to above.

Other than as permitted in any relevant BMJ Author's Self Archiving Policies, I confirm this Work has not been accepted for publication elsewhere, is not being considered for publication elsewhere and does not duplicate material already published. I confirm all authors consent to publication of this Work and authorise the granting of this licence.

For peer review only

Mental Wellbeing of Health Care Workers in Lusaka, Zambia During the COVID-19 Pandemic

Sandra Simbeza[1], Jacob Mutale[1], Musunge Mulabe[1], Lazarus Jere[1], Chama Bukankala[1], , Izukanji Sikazwe[1], Carolyn Bolton Moore[1,3], Aaloke Mody[4], Elvin H. Geng[4], Anjali Sharma[1], Laura K. Beres[2†], Jake M. Pry[1,5§†]

[1]Centre for infectious Disease Research in Zambia, Lusaka Zambia
[2]Department of International Health, Johns Hopkins Bloomberg School of Public Health, Baltimore, MD USA
[3]School of Medicine, University of Alabama, Birmingham AL, USA
[4]School of Medicine, Washington University in St. Louis, MO USA
[5]Department of Public Health Sciences, School of Medicine, University of California, Davis, CA, USA

[†]Authors contributed equally to manuscript

[§] Corresponding author: Jake M. Pry

Jake M. Pry, PhD, MPH
Centre for Infectious Disease Research Zambia
Plot 34620 Danny Pule Road, Mass Media
Lusaka, Zambia 10101
jake.pry@cidrz.org | jmpry@ucdavis.edu
Phone: +1 936 661 6885

Keywords: Health Care Workers, Depression, COVID-19, PHQ-9

For peer review only - http://bmjopen.bmj.com/site/about/guidelines.xhtml

Abstract

Objectives: We sought to assess mental health among HCWs in the context of COVID-19 across 24 health facilities in Lusaka Province, Zambia.

Methods: We implemented the, well-validated, Patient Health Questionnaire (PHQ-9) to assess depression through a cross-sectional study of HCWs recruited though convenience sampling during 11 August - 15 October 2020 during the second wave of the COVID-19 epidemic in Zambia. Potential participants were invited from 24 government-funded health facilities in Lusaka Province, Zambia. We used mixed-effects, adjusted Poisson regression to estimate the marginal probability of HCW experiencing depression that may warrant intervention (PHQ-9 score ≥5) by healthcare facility.

Results: We collected PHQ-9 survey responses from 713 professional and lay HCWs. Overall, 334 (46.8%, 95% CI: 43.1, .50.6%) HCWs recorded a PHQ-9 score ≥5, indicating the need for further screening and assessment for mental health disorders, as well as preventative interventions, to minimize the possibility of poor health outcomes. We identified significant heterogeneity across facilities and observed a greater proportion of HCWs in facilities providing COVID-19 testing and treatment services with symptoms of depression.

Conclusions: Mental wellbeing may be a concern for a large proportion of HCW in Zambia. Further work to understand the magnitude and etiologies of mental health disorders amongst HCW in the public sector is needed to design effective interventions to meet the need for mental health support.

For peer review only - http://bmjopen.bmj.com/site/about/guidelines.xhtml

BMJ Open Questions:

Strengths and Limitations of this study

- These data represent important insight regarding the state of mental wellness among a large sample of healthcare workers during a public health crisis in a resource limited setting.
- Mental wellness, like depression, is not documented well in resource limited settings and we measure here mental wellness in a high-risk group of healthcare workers during the COVID-19 pandemic.
- An important limitation is lack of data on demographics of healthcare worker respondents due to concern about stigma around mental wellness and potential to identify individuals given inclusion of clinics with small staff.
- We include a large number (N=24) of facilities to appreciate the heterogeneity in symptoms of depression among mental health in the sampled facilities in Lusaka Province of Zambia.

For peer review only

Introduction

Coronavirus disease 2019 (COVID-19) has caused substantial global health hardship and healthcare workers (HCWs) position on the frontlines of the public health response, places them at great risk for negative effects on health and wellbeing. In addition to excess occupational hazard of contracting COVID-19, their experience as care givers increases risk of developing mental health disorders such as anxiety, depression, trauma, insomnia, and stress, which may lead to poor physical and psychological wellbeing [1]. A healthy workforce is critical to effectively managing and mitigating COVID-19, as well as providing continuity of high-quality care for other chronic and acute health conditions [2, 3]. Even prior to COVID-19, countries in sub-Saharan Africa faced limited medical infrastructure, supplies, and an over-burdened workforce, which challenged the provision of quality healthcare [4, 5]. These challenges, along with pandemic-sensitive barriers such as limited access to personal protective equipment (PPE) further exacerbated HCW stress and vulnerability. While HCWs in resource limited settings have demonstrated resilience, poor mental health is likely to compromise their ability to make decisions**,** as well as impact patient interactions [6]. Mental health services, like other health resources, are limited, with few trained mental health providers[7]. Understanding mental health and wellbeing among HCWs can catalyze interventions to provide treatment and improve the healthcare facility environment for the HCWs and patients [7].

Several studies have been conducted to assess mental wellness impact of the pandemic amongst HCWs however, the majority of these studies focus on the continent of Asia and very little data is available for HCWs in Africa [8, 9]. Limited data from Kenya and Ethiopia provides evidence that the prevalence of mental disorders such as depression, insomnia and stress were higher among those HCWs caring for patients with COVID-19, or in areas of higher infection prevalence compared to those working with non-COVID-19 patients or less [1, 10-14]. The most prevalent reported mental health conditions among health care workers are depression, insomnia, and anxiety [15]. Characterization of the state of mental wellness during the COVID-19 pandemic in Sub-Saharan African countries among HCWs, specifically in Zambia remains incomplete [10, 16].

As part of a larger patient-centered care study, we collected facility-level measures to understand the context of care at 24 study sites. As a part of establishing care context we assessed mental health, specifically depression, using the nine item Patient Health Questionnaire (PHQ-9) in the

For peer review only - http://bmjopen.bmj.com/site/about/guidelines.xhtml

context of COVID-19 among HCWs in Lusaka Province, Zambia from 11 August 2020 to 15 October 2020 [17].

Methods

Setting

This cross-sectional study is nested within a larger study, Person Centered Public Health for HIV Treatment in Zambia (PCPH), a cluster-randomized trial to assess HIV care and outcomes running from August 2018 to November 2021 across 24 government-funded health facilities in Lusaka Province (Pan African Clinical Trial Registry number: PACTR202101847907585). All participating facilities offered HIV care services and varied in size and location. Facilities were assigned codes to protect the identity of the HCW participants. As a part of the PCPH package adaptation to the COVID-19 pandemic, we implemented the PHQ-9 to screen for presence and severity of depression. The PHQ-9 has been previously used in Zambia to screen for likelihood of depression [20], and has been validated in Tanzania and South Africa which are similar settings as Zambia [18, 19]. The PHQ-9 instrument was translated from English into Nyanja and Bemba, the most commonly spoken Zambian languages in Lusaka Province, where the survey was conducted [20].

Study Population

We developed a cohort of HCWs that primarily provide HIV care at one of the 24 health facilities in the PCPH study. This was done by compiling all HCW's contact details from the HCWs who had, at the time of the introduction of the PHQ-9 measure to the study, participated in the HCW survey component of the PCPH study. The PCPH sample was comprised of both professional and lay HCWs including nurses, pharmacists, treatment supporters, midwives, medical doctors, radiographers, and general workers. To augment participation in the PHQ-9 data collection, we expanded the PHQ-9 sample to include any HCW willing to participate and had ≥6 months working experience at the study facility. We informed the facility in-charge of the PHQ-9 study opportunity who communicated it to staff members.

Measurements

For peer review only - http://bmjopen.bmj.com/site/about/guidelines.xhtml

99 Trained study research assistants then visited the facility and discussed the study opportunity in-person with available staff members. Those who expressed interest were screened for eligibility, offered the opportunity to verbally consent and with immediate participation. Depending on the participant preference, the standard PHQ-9 survey was self-administered on paper or surveyor-administered by trained study research assistants in the participant's preferred language. Potentially identifying information such as age, sex, and HCW cadre were not collected from respondents to protect privacy.

Analysis

We followed the standard, 27-point scoring system for the PHQ-9 to identify participants with scores consistent with minimal (0-4), mild (5-9), moderate (10-14), moderately severe (15-19), and severe depression (20-27) [18]. Our main outcome was mild depression or great (PHQ-9 score >=5) as this level of depression warrants additional clinical follow-up. We developed frequency tables and used a bar graphs to illustrate the distribution of PHQ-9 scores by healthcare facility. We developed scatter plots of adjusted marginal probability with 95% confidence intervals to illustrate the probability that a HCW will have a PHQ-9 score ≥5 by healthcare facility. We used mixed effects Poisson regression to estimate prevalence ratios for those with mild depression allowing random effects at the facility level and measured fixed effects for month of survey and clinic size category. Facilities were categorized by client population estimates as small (<40,000 clients), medium (40,000-100,000 clients), and large (>100,000 clients).

Patient and Public Involvement

The parent study, focused primarily on improving the patient experience at routine clinic visits through HCW mentorship and support including assessments of mental wellness through administration of the PHQ-9 tool, the results of which are captured here. The research approach and content is based on previous research, human-centered design workshops, and stakeholder collaborations [21-27]. We invited HCWs from each of the study facilities to attend a human-centered design workshop with expert direction from a professional consulting group in Washington DC. The results of this workshop were incorporated into the patient experience feedback summary design for the study clinics and the implementation of trained patient assessment was modified to improve transparency and acceptability. Patients were not directly

For peer review only - http://bmjopen.bmj.com/site/about/guidelines.xhtml

involved in the design, nor the recruitment of this sub-study assessing the mental wellness of HCWs. The findings of this study as well as the parent study will be shared with the Zambian Ministry of Health as well as the study facilities.

Ethics

This study was approved as part of the larger PCPH study by the University of Zambia Biomedical Ethics Committee and the University of Alabama at Birmingham Institution Review Board, and the National Health Research Authority in Zambia. We obtained waiver of written informed consent and obtained verbal consent from participants prior to administering the survey.

Results

A total of 713 HCWs from 24 facilities across Lusaka and Chongwe districts in the Lusaka Province were included in the analysis dataset (**Table 1**). Of the 713 responses, 231 (32.4%) reported mild depression (PHQ-9 score 5-9) and 81 (11.4%) reported moderate depression of (PHQ-9 score 10-14). The majority (69.6%) of the PHQ-9 survey responses were collected in September 2020 (**Table 1**). Responses for PHQ-9 questions were largely complete with <1% responses missing.

Table 1: Participant characteristics

Factor	Level	n (%)
N		713
Facility	1	30 (4.2)
	2	30 (4.2)
	3	30 (4.2)
	4	30 (4.2)
	5	30 (4.2)
	6	30 (4.2)
	7	30 (4.2)
	8	30 (4.2)
	9	30 (4.2)
	10	30 (4.2)
	11	30 (4.2)
	12	27 (3.8)

For peer review only - http://bmjopen.bmj.com/site/about/guidelines.xhtml

For peer review only

	13	27 (3.8)
	14	30 (4.2)
	15	30 (4.2)
	16	30 (4.2)
	17	30 (4.2)
	18	30 (4.2)
	19	30 (4.2)
	20	30 (4.2)
	21	30 (4.2)
	22	30 (4.2)
	23	29 (4.1)
	24	30 (4.2)
Clinic Population (category)	Small	147 (20.6)
	Medium	269 (37.7)
	Large	297 (41.7)
PHQ-9 Score Category	minimal	379 (53.2)
	mild	231 (32.4)
	moderate	81 (11.4)
	moderately severe	17 (2.4)
	severe	5 (0.7)
Month of Survey	August 2020	205 (28.8)
	September 2020	496 (69.6)
	October 2020	12 (1.7)

Note: PHQ-9 – nine-item patient health questionnaire.

A total of 81 (11.4) respondents had a PHQ-9 score corresponding to moderate depression across all but one facility, 17 (2.4%) respondents had PHQ-9 scores consistent with moderately severe depression across four facilities and five (0.7%) HCWs had PHQ-9 scores consistent with severe depression across five different facilities (**Fig 1, Table 2**).

Table 2: Proportion of the analysis population by PHQ-9 score category with 95% confidence intervals (N=713)

Variable	*n*	*Proportion (%)*	*95% CI*
≥5 PHQ-9 score	334	46.8	(43.1, 50.6)
Mild	231	32.4	(29.0, 36.0)
Moderate	81	11.4	(9.1, 13.9)
Moderate-severe	17	2.4	(1.4, 3.8)
Severe	5	0.7	(0.2, 1.6)

Note: CI – confidence interval; PHQ-9 – nine-item patient health questionnaire.

For peer review only

For peer review only - http://bmjopen.bmj.com/site/about/guidelines.xhtml

Though we observed facility-level mental health heterogeneity in proportion of minimal and mild depression scores across clinics, the proportion of scores consistent with moderate depression remain relatively stable across facilities (**Fig 1A).**

Figure 1: A) Stacked bar chart of proportion population by PHQ-9 score category by healthcare facility B) Stacked bar chart of proportion population by PHQ-9 score by facility population size category

Mixed effects adjusted Poisson regression model did not reveal clinic size or month of survey as a significant predictor of PHQ-9 score ≥5 (**Table 3**).

Table 3: Adjusted Poisson regression results (N=713)

Covariate	*level*	*aPR*	*95% CI*	*p-value*
Clinic Population (category)	small	1.12	(0.95, 1.33)	0.170
	medium	1.00 (ref)	ref	ref
	large	1.04	(0.81, 1.33)	0.763
Month of Survey	August	1.00 (ref)	ref	ref
	September	1.14	(0.94, 1.38)	0.193
	October	0.98	(0.54, 1.79)	0.945

Note: adjusted for survey week and facility

We illustrate significant heterogeneity in the marginal probability of experiencing greater than minimal depression across facilities (**Fig 2**). Notably higher marginal probability of HCWs with PHQ-9 score ≥5 was observed in both COVID-19 treatment centers and non-COVID-19 treatment centers, facilities 4 and 16.

Figure 2: Marginal probability of healthcare worker with >minimal depression per PHQ-9 score by clinic.

Note: PHQ-9 – nine-item patient health questionnaire.

For peer review only - http://bmjopen.bmj.com/site/about/guidelines.xhtml

Discussion

We found that a large proportion of the HCW population had a PHQ-9 score ≥5 indicating need for some level of mental wellness follow-up 46.8% (95% CI: 43.1, 50.6%). Variation in depression outcomes ranged from 82.7% (95% CI: 68.5, 96.9%) at one of the two large COVID-19 treatment facilities to 28.4% (95% CI: 13.0, 43.9%) at a medium-sized healthcare facility. Differences in clinic characteristics, such as size of catchment population and location could contribute to the variations in depression outcomes among the facilities.

Past studies show that frontline HCW working in clinics, considered to be highly infectious, such as COVID-19 treatment centers, were more prone to developing depression and other mental disorders than their counterparts in other departments [10]. This is in consistent with the results of our study, which shows that HCWs working at COVID-19 treatment facilities had a marginal probability of experiencing mild to moderately severe depression. In addition, results of our study demonstrate that HCWs have experienced symptoms of depression during the onset of the COVID-19 pandemic in Zambia, which is consistent with the findings of similar studies using the PHQ-9 in other parts of the world where a pooled prevalence of mild depression was found to be 36.1% (95% confidence interval [CI], 31.3%-41.0%) [28]. Studies conducted in Zambia among nurses and midwives, and in Ethiopia among different cadres of HCWs shows that mental disorders which include depression are prevalent among HCWs during the COVID-19 pandemic [29]. Similarly, globally studies, indicate, that the risk of HCWs developing mental health disorders during the pandemic exist [3, 28, 29]. The difficult conditions to which HCWs may be exposed including extended working hours, risk of exposure to the disease, increased workload exposure to the disease, concerns about transmitting the infection to their family members, limited resources to care for their patients, may exacerbate or initialize mental wellbeing issues leading to symptoms of depression [30, 31].

The heterogeneity in proportions of HCWs with depression may be driven by facility size, where reduced resources at smaller facilities including, but not limited to, personal protective equipment (PPE), hand hygiene station/station supplies at clinic entrance, and less access to knowledge about the COVID-19 response in Zambia compared to larger facilities in urban Lusaka. While the response to the COVID-19 pandemic was standardized, to a certain extent, by guidance from the

For peer review only - http://bmjopen.bmj.com/site/about/guidelines.xhtml

provincial and zonal levels, the culture and leadership unique to each facility might have played a key role in the healthcare worker experience, contributing to PHQ-9 score distribution differences across facilities.

As we continue to recognize the mental health services gap across many populations in resource-limited settings we give evidence here to support prioritization of healthcare workers, especially during public health shocks/emergencies like that presented by COVID-19. Mental wellness challenges among HCWs could lead to poor health outcomes for the HCW workforce and have a sort of "knock-on" effect negatively impacting the quality of care provided to patients [32, 33]. Interventions like Friendship Bench piloted in neighboring Zimbabwe designed to encourage positive coping mechanism among HCWs and build a working environment that provides empathy and compassion towards staff may be an efficient option to provide mental wellness support [34, 35]. Furthermore, system-based interventions should also be encouraged such as change in working culture and reduction in possible system contributors to HCW stress that could lead to depression. Increasing mobile technology availability may further allow for the use of mobile health (mHealth) based mental wellness services leveraging the framework presented by Osei for low- and middle-income countries [36, 37]. Low-cost intervention packages used for patients can be adapted for HCWs and integrated into system-based interventions. They include routine screening for early detection, mental wellness education, problem solving and use of anti-depressants, cognitive behavior therapy which can be delivered successfully by trained lay HCWs [38, 39].

Limitations

This study had some potential limitations. Firstly, we did not collect participants demographics such as age, sex, marital status, and sex. As such we were not able to adjust for these co- founders. Accounting for these would help make associations in outcomes of depression. Secondly, we employed convenience sampling in selecting participants, this sampling approach may limit representativeness of study the results to the wider population of HCWs. Finally, without pre-COVID 19 estimates of depression among healthcare workers in Lusaka, we are not able to show association between mild-severe depression with the pandemic however, we show that during the pandemic that depression was high and attention to this population is justified to ensure a healthy

For peer review only - http://bmjopen.bmj.com/site/about/guidelines.xhtml

HCW workforce. Additionally, though potentially highest than a non-pandemic baseline, these results may be useful as subsequent measures of depression and mental wellness are collected among HCWs.

Conclusions

Depression is a common public health problem; our study demonstrates that HCWs in Zambia may suffer from a high prevalence of depressive symptoms. Routine mental health wellness is important to better understand the role that the COVID-19 pandemic may have had on depression among HCWS in Zambia. Furthermore, support for HCW mental wellness can serve to accelerate destigmatizing mental health issues and improve the quality of care provided across healthcare centers in Zambia.

For peer review only - http://bmjopen.bmj.com/site/about/guidelines.xhtml

Competing interests

Authors do not have competing interests to report.

Authors' contributions

SS: Data Collection, Data Framing, Manuscript Writing

JM: Data Collection, Data Curation, Data Analysis

MM: Data Collection, Data Curation

LJ: Data Framing

CB: Data Framing

KS: Conceptualization

IS: Conceptualization

CBM: Conceptualization

AM: Analysis Review, Manuscript Review/Editing

EHG: Conceptualization

AS: Conceptualization, Manuscript Review/Editing

LKB: Conceptualization, Data Analysis, Data Framing, Manuscript Writing

JMP: Data Analysis Lead, Data Framing, Manuscript Writing

Author information

[not aware of anything needed here]

Acknowledgements

We would like to thank the Zambian Ministry of Health for their leadership to ensure those in care continue to receive life-saving HIV treatment. We would also like to thank the healthcare workers for their participation in the PHQ-9 assessment as well as their faithful delivery of care to patients in the face of the many challenges presented by the COVID-19 pandemic. To the clients seen at our study facilities we would like to extend sincere thanks for your cooperation and patience.

Funding

For peer review only

For peer review only - http://bmjopen.bmj.com/site/about/guidelines.xhtml

Research reported in this publication was supported by the Bill & Melinda Gates Foundation grant number OPP1166485, the National Institute Of Mental Health of the National Institutes of Health under award number F31MH109378 (L.K.B.), and the National Institute of Allergy and Infectious Diseases of the National Institutes of Health under award number K24 AI134413 (E.G.).

Disclaimer

None

List of abbreviations [Optional]

ART: antiretroviral therapy

CI- Confidence Interval

COVID-19: Coronavirus Disease 2019

HCW: Health Care Worker

PCPH: Person- Centered Public Health for HIV Treatment in Zambia

PHQ-9: Patient Health Questionnaire 9

Data Sharing

Data used for this analysis is available through Dryad data repository (doi:10.25338/B8S646)

References

1. Robertson L, Maposa I, Somaroo H, Johnson O. Mental health of healthcare workers during the COVID-19 outbreak: A rapid scoping review to inform provincial guidelines in South Africa. South African Medical Journal. 2020;110(10):1010-9.

2. Gupta S, Sahoo S. Pandemic and mental health of the front-line healthcare workers: a review and implications in the Indian context amidst COVID-19. General Psychiatry. 2020;33(5).

3. Saragih ID, Tonapa SI, Saragih IS, Advani S, Batubara SO, Suarilah I, et al. Global Prevalence of Mental Health Problems among healthcare workers during The Covid-19 Pandemic: A Systematic Review and Meta-Analysis. International Journal of Nursing Studies. 2021:104002.

4. Ayanore MA, Amuna N, Aviisah M, Awolu A, Kipo-Sunyehzi DD, Mogre V, et al. Towards resilient health systems in sub-saharan africa: A systematic review of the english language literature on health workforce, surveillance, and health governance issues for health systems strengthening. Annals of global health. 2019;85(1).

5. Semo B-w, Frissa SM. The mental health impact of the COVID-19 pandemic: implications for sub-Saharan Africa. Psychology Research and Behavior Management. 2020;13:713.

For peer review only - http://bmjopen.bmj.com/site/about/guidelines.xhtml

6. Med S. Serious Health Workforce Shortage in Zambia: Solidar Med; 2020 [updated 22 December 2020; cited 2022 16 January 2022]. Available from: https://www.solidarmed.ch/en/news/shortage-skilled-workers-health-system-zambia.

7. Munakampe MN. Strengthening mental health systems in Zambia. International journal of mental health systems. 2020;14(1):1-9.

8. Deng D, Naslund JA. Psychological impact of COVID-19 Pandemic on frontline health workers in low-and middle-income countries. Harvard public health review (Cambridge, Mass). 2020;28.

9. Moitra M, Rahman M, Collins PY, Gohar F, Weaver M, Kinuthia J, et al. Mental health consequences for healthcare workers during the COVID-19 pandemic: a scoping review to draw lessons for LMICs. Frontiers in psychiatry. 2021;12:22.

10. Shah J, Monroe-Wise A, Talib Z, Nabiswa A, Said M, Abeid A, et al. Mental health disorders among healthcare workers during the COVID-19 pandemic: a cross-sectional survey from three major hospitals in Kenya. BMJ open. 2021;11(6):e050316.

11. Chersich MF, Gray G, Fairlie L, Eichbaum Q, Mayhew S, Allwood B, et al. COVID-19 in Africa: care and protection for frontline healthcare workers. Global Health. 2020;16(1):46. Epub 2020/05/18. doi: 10.1186/s12992-020-00574-3. PubMed PMID: 32414379; PubMed Central PMCID: PMCPMC7227172.

12. Yitayih Y, Mekonen S, Zeynudin A, Mengistie E, Ambelu A. Mental health of healthcare professionals during the early stage of the COVID-19 pandemic in Ethiopia. BJPsych Open. 2020;7(1):e1. Epub 2020/12/02. doi: 10.1192/bjo.2020.130. PubMed PMID: 33256883; PubMed Central PMCID: PMCPMC7844150.

13. Jemal K, Deriba BS, Geleta TA, Tesema M, Awol M, Mengistu E, et al. Self-Reported Symptoms of Depression, Anxiety, and Stress Among Healthcare Workers in Ethiopia During the COVID-19 Pandemic: A Cross-Sectional Study. Neuropsychiatr Dis Treat. 2021;17:1363-73. Epub 2021/05/15. doi: 10.2147/NDT.S306240. PubMed PMID: 33986596; PubMed Central PMCID: PMCPMC8110274.

14. Asnakew S, Amha H, Kassew T. Mental Health Adverse Effects of COVID-19 Pandemic on Health Care Workers in North West Ethiopia: A Multicenter Cross-Sectional Study. Neuropsychiatr Dis Treat. 2021;17:1375-84. Epub 2021/05/18. doi: 10.2147/NDT.S306300. PubMed PMID: 33994787; PubMed Central PMCID: PMCPMC8113010.

15. Sahebi A, Nejati-Zarnaqi B, Moayedi S, Yousefi K, Torres M, Golitaleb M. The prevalence of anxiety and depression among healthcare workers during the COVID-19 pandemic: An umbrella review of meta-analyses. Prog Neuropsychopharmacol Biol Psychiatry. 2021;107:110247. Epub 2021/01/22. doi: 10.1016/j.pnpbp.2021.110247. PubMed PMID: 33476692; PubMed Central PMCID: PMCPMC7817526.

16. Elbay RY, Kurtulmuş A, Arpacıoğlu S, Karadere E. Depression, anxiety, stress levels of physicians and associated factors in Covid-19 pandemics. Psychiatry research. 2020;290:113130.

17. Kroenke K, Spitzer RL, Williams JB. The PHQ-9: validity of a brief depression severity measure. Journal of general internal medicine. 2001;16(9):606-13. doi: 10.1046/j.1525-1497.2001.016009606.x. PubMed PMID: 11556941.

18. Kroenke K, Spitzer RL, Williams JB. The PHQ-9: validity of a brief depression severity measure. Journal of general internal medicine. 2001;16(9):606-13.

19. Kroenke K, Spitzer RL, Williams JB, Löwe B. The patient health questionnaire somatic, anxiety, and depressive symptom scales: a systematic review. General hospital psychiatry. 2010;32(4):345-59.

For peer review only - http://bmjopen.bmj.com/site/about/guidelines.xhtml

20. Sawka KS, Mbewe C, Josephat D, Ngulube E. The Senga of Zambia: Linguistic Survey Report with Recommendations for Literature Development Strategy. SIL International: 2021.
21. Beres LK, Simbeza S, Holmes CB, Mwamba C, Mukamba N, Sharma A, et al. Human-centered design lessons for implementation science: improving the implementation of a patient-centered care intervention. Journal of acquired immune deficiency syndromes (1999). 2019;82(3):S230.
22. Mody A, Tram KH, Glidden DV, Eshun-Wilson I, Sikombe K, Mehrotra M, et al. Novel Longitudinal Methods for Assessing Retention in Care: a Synthetic Review. Current HIV/AIDS Reports. 2021;18(4):299-308.
23. Sikombe K, Mody A, Kadota J, Pry JJ, Simbeza S, Eshun-Wilson I, et al. Understanding patient transfers across multiple clinics in Zambia among HIV infected adults. PloS one. 2020;15(11):e0241477.
24. Holmes CB, Sanne I. Changing models of care to improve progression through the HIV treatment cascade in different populations. Current Opinion in HIV and AIDS. 2015;10(6):447-50.
25. Holmes CB, Sikazwe I, Sikombe K, Eshun-Wilson I, Czaicki N, Beres LK, et al. Estimated mortality on HIV treatment among active patients and patients lost to follow-up in 4 provinces of Zambia: Findings from a multistage sampling-based survey. PLoS Med. 2018;15(1):e1002489. Epub 2018/01/13. doi: 10.1371/journal.pmed.1002489. PubMed PMID: 29329301; PubMed Central PMCID: PMCPMC5766235.
26. Roy M, Czaicki N, Holmes C, Chavan S, Tsitsi A, Odeny T, et al. Understanding Sustained Retention in HIV/AIDS Care and Treatment: a Synthetic Review. Current HIV/AIDS Reports. 2016:1-9.
27. Mwamba C, Sharma A, Mukamba N, Beres L, Geng E, Holmes CB, et al. 'They care rudely!': resourcing and relational health system factors that influence retention in care for people living with HIV in Zambia. BMJ Glob Health. 2018;3(5):e001007. Epub 2018/11/30. doi: 10.1136/bmjgh-2018-001007. PubMed PMID: 30483408; PubMed Central PMCID: PMCPMC6231098.
28. Li Y, Scherer N, Felix L, Kuper H. Prevalence of depression, anxiety and post-traumatic stress disorder in health care workers during the COVID-19 pandemic: A systematic review and meta-analysis. PloS one. 2021;16(3):e0246454.
29. Mulatu HA, Tesfaye M, Woldeyes E, Bayisa T, Fisseha H, Kassu RA. The prevalence of common mental disorders among healthcare professionals during the COVID-19 pandemic at a tertiary Hospital in Addis Ababa, Ethiopia. Journal of Affective Disorders Reports. 2021;6:100246. doi: https://doi.org/10.1016/j.jadr.2021.100246.
30. Mokhtari R, Moayedi S, Golitaleb M. COVID-19 pandemic and health anxiety among nurses of intensive care units. International journal of mental health nursing. 2020;29(6):1275.
31. Pfefferbaum B, North CS. Mental health and the Covid-19 pandemic. New England Journal of Medicine. 2020;383(6):510-2.
32. Saasa Y. An Assessment on the Role of Human Rights and Governance in Health Service Delivery 2019.
33. Kola L, Kohrt BA, Hanlon C, Naslund JA, Sikander S, Balaji M, et al. COVID-19 mental health impact and responses in low-income and middle-income countries: reimagining global mental health. The Lancet Psychiatry. 2021;8(6):535-50. doi: https://doi.org/10.1016/S2215-0366(21)00025-0.

For peer review only

For peer review only - http://bmjopen.bmj.com/site/about/guidelines.xhtml

34. Abraham A, Chaabna K, Doraiswamy S, Bhagat S, Sheikh J, Mamtani R, et al. Depression among healthcare workers in the Eastern Mediterranean Region: a systematic review and meta-analysis. Human resources for health. 2021;19(1):1-18.
35. Wallén A, Eberhard S, Landgren K. The Experiences of Counsellors Offering Problem-Solving Therapy for Common Mental Health Issues at the Youth Friendship Bench in Zimbabwe. Issues in Mental Health Nursing. 2021;42(9):808-17. doi: 10.1080/01612840.2021.1879977.
36. Osei E, Mashamba-Thompson TP. Mobile health applications for disease screening and treatment support in low-and middle-income countries: A narrative review. Heliyon. 2021;7(3):e06639. Epub 2021/04/20. doi: 10.1016/j.heliyon.2021.e06639. PubMed PMID: 33869857; PubMed Central PMCID: PMCPMC8035664.
37. Mutimushi PM, Mupenda BM, Manda PM, Nankonde N, Banda B, Mung'omba K, et al. ZICTA 2019 Annual Report. https://www.zicta.zm/storage/posts/attachments/oiBT4oMnpJoN3dgHKpvByoDmNXq5ZDYtp3sxVYM3.pdf: Zambia Information and Communications Technology Authority (ZICTA), 2019.
38. Chibanda D, Mesu P, Kajawu L, Cowan F, Araya R, Abas MA. Problem-solving therapy for depression and common mental disorders in Zimbabwe: piloting a task-shifting primary mental health care intervention in a population with a high prevalence of people living with HIV. BMC public health. 2011;11(1):1-10.
39. Patel V, Simon G, Chowdhary N, Kaaya S, Araya R. Packages of care for depression in low-and middle-income countries. PLoS medicine. 2009;6(10):e1000159.

Supplement:

Figure S1: Histogram of total PHQ-9 scores among study participants

For peer review only - http://bmjopen.bmj.com/site/about/guidelines.xhtml

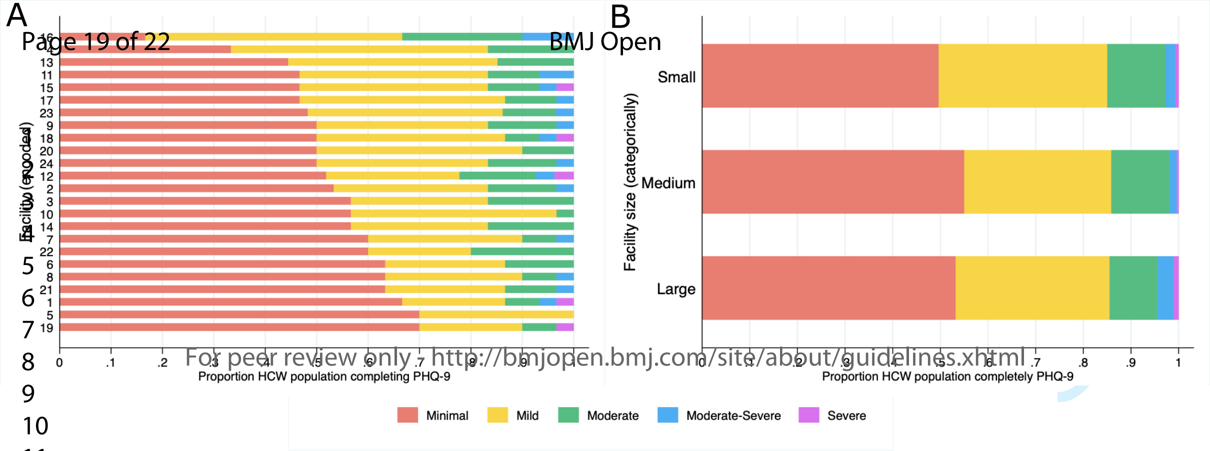
A
Facility (encoded)
16
4
13
11
15
17
23
9
18
20
24
12
2
3
10
14
7
22
6
8
21
1
5
19
0 .1 .2 .3 .4 .5 .6 .7 .8 .9 1
Proportion HCW population completing PHQ-9
B
Facility size (categorically)
Small
Medium
Large
0 .1 .2 .3 .4 .5 .6 .7 .8 .9 1
Proportion HCW population completely PHQ-9
Minimal
Mild
Moderate
Moderate-Severe
Severe

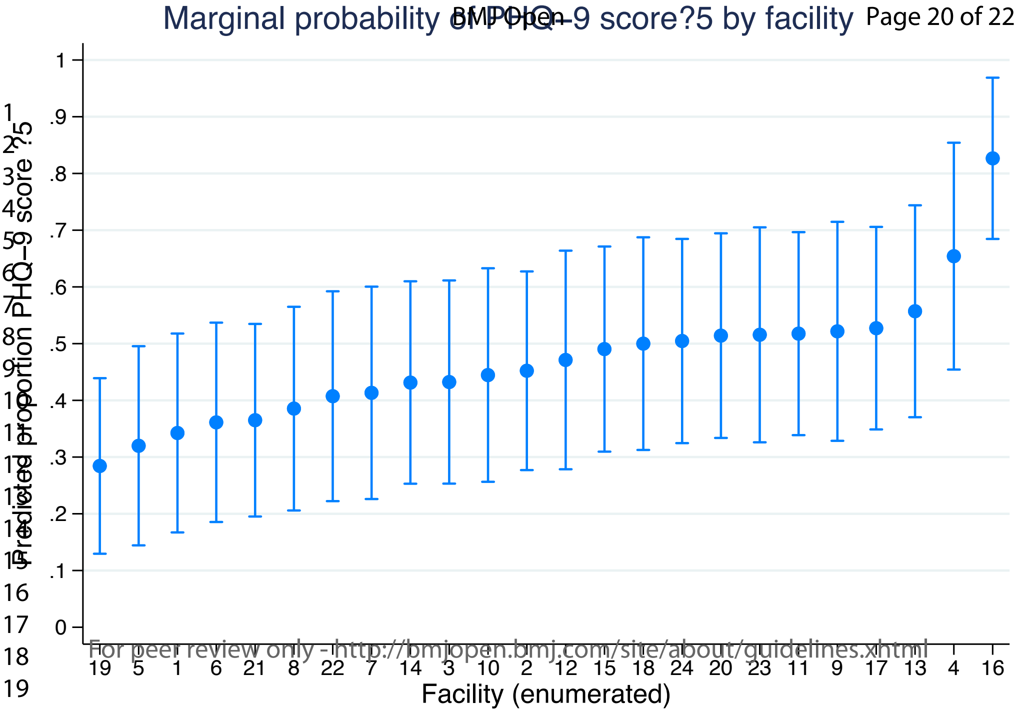

For peer review only - http://bmjopen.bmj.com/site/about/guidelines.xhtml

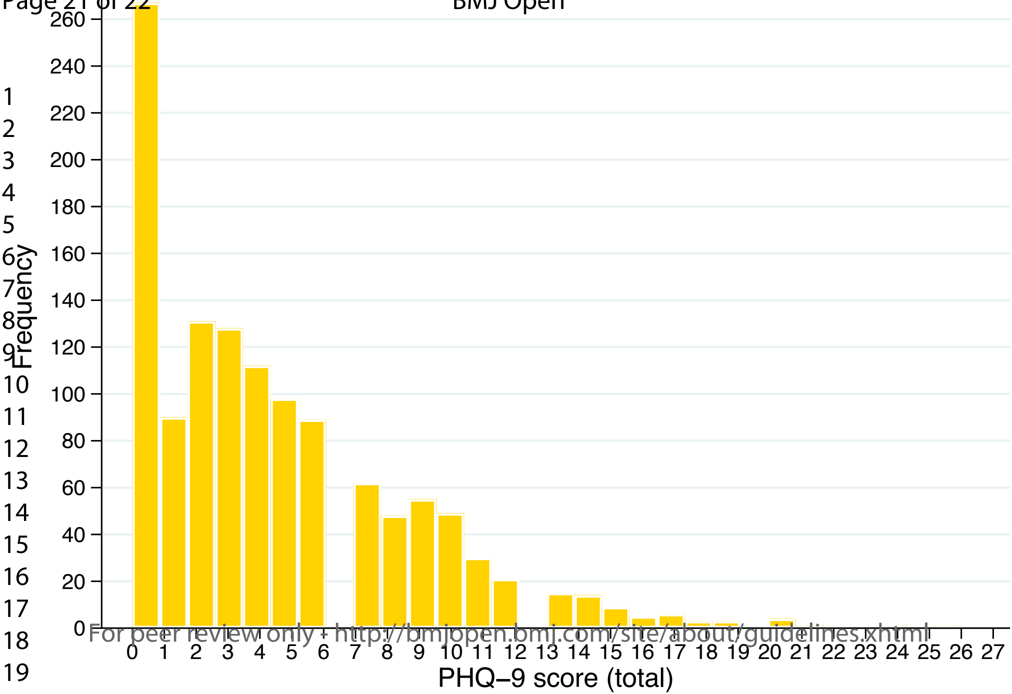

For peer review only - http://bmjopen.bmj.com/site/about/guidelines.xhtml

STROBE Statement—Checklist of items that should be included in reports of ***cohort studies***

	Item No	Recommendation	Page No
**Title and abstract**	1	(*a*) Indicate the study's design with a commonly used term in the title or the abstract	2
		(*b*) Provide in the abstract an informative and balanced summary of what was done and what was found	2
**Introduction**			
Background/rationale	2	Explain the scientific background and rationale for the investigation being reported	4
Objectives	3	State specific objectives, including any prespecified hypotheses	4, 5
**Methods**			
Study design	4	Present key elements of study design early in the paper	5
Setting	5	Describe the setting, locations, and relevant dates, including periods of recruitment, exposure, follow-up, and data collection	5
Participants	6	(*a*) Give the eligibility criteria, and the sources and methods of selection of participants. Describe methods of follow-up	5, 6
		(*b*) For matched studies, give matching criteria and number of exposed and unexposed	NA
Variables	7	Clearly define all outcomes, exposures, predictors, potential confounders, and effect modifiers. Give diagnostic criteria, if applicable	5, 6
Data sources/ measurement	8*	For each variable of interest, give sources of data and details of methods of assessment (measurement). Describe comparability of assessment methods if there is more than one group	5, 6
Bias	9	Describe any efforts to address potential sources of bias	5, 6
Study size	10	Explain how the study size was arrived at	5
Quantitative variables	11	Explain how quantitative variables were handled in the analyses. If applicable, describe which groupings were chosen and why	5, 6
Statistical methods	12	(*a*) Describe all statistical methods, including those used to control for confounding	6
		(*b*) Describe any methods used to examine subgroups and interactions	6
		(*c*) Explain how missing data were addressed	6
		(*d*) If applicable, explain how loss to follow-up was addressed	NA
		(*e*) Describe any sensitivity analyses	6
**Results**			
Participants	13*	(a) Report numbers of individuals at each stage of study—eg numbers potentially eligible, examined for eligibility, confirmed eligible, included in the study, completing follow-up, and analysed	7, 8
		(b) Give reasons for non-participation at each stage	NA
		(c) Consider use of a flow diagram	NA
Descriptive data	14*	(a) Give characteristics of study participants (eg demographic, clinical, social) and information on exposures and potential confounders	7, 8
		(b) Indicate number of participants with missing data for each variable of interest	7, 8
		(c) Summarise follow-up time (eg, average and total amount)	NA
Outcome data	15*	Report numbers of outcome events or summary measures over time	7, 8

For peer review only

Main results	16	(*a*) Give unadjusted estimates and, if applicable, confounder-adjusted estimates and their precision (eg, 95% confidence interval). Make clear which confounders were adjusted for and why they were included	7, 8
		(*b*) Report category boundaries when continuous variables were categorized	7, 8
		(*c*) If relevant, consider translating estimates of relative risk into absolute risk for a meaningful time period	NA
Other analyses	17	Report other analyses done—eg analyses of subgroups and interactions, and sensitivity analyses	9
**Discussion**			
Key results	18	Summarise key results with reference to study objectives	10
Limitations	19	Discuss limitations of the study, taking into account sources of potential bias or imprecision. Discuss both direction and magnitude of any potential bias	11, 12
Interpretation	20	Give a cautious overall interpretation of results considering objectives, limitations, multiplicity of analyses, results from similar studies, and other relevant evidence	9, 10
Generalisability	21	Discuss the generalisability (external validity) of the study results	9, 10
**Other information**			
Funding	22	Give the source of funding and the role of the funders for the present study and, if applicable, for the original study on which the present article is based	13, 14

*Give information separately for exposed and unexposed groups.

Note: An Explanation and Elaboration article discusses each checklist item and gives methodological background and published examples of transparent reporting. The STROBE checklist is best used in conjunction with this article (freely available on the Web sites of PLoS Medicine at http://www.plosmedicine.org/, Annals of Internal Medicine at http://www.annals.org/, and Epidemiology at http://www.epidem.com/). Information on the STROBE Initiative is available at http://www.strobe-statement.org.

For peer review only

A cross-sectional study to assess depression among health care workers in Lusaka, Zambia during the COVID-19 pandemic

Journal:	*BMJ Open*
Manuscript ID	bmjopen-2022-069257.R1
Article Type:	Original research
Date Submitted by the Author:	29-Dec-2022
Complete List of Authors:	Simbeza, Sandra; Center for Infectious Disease Research in Zambia<br>Mutale, Jacob; Center for Infectious Disease Research in Zambia, Implementation Science Unit<br>Mulabe, Musunge; Center for Infectious Disease Research in Zambia<br>Jere, Lazarus; Center for Infectious Disease Research in Zambia<br>Bukankala, Chama; Center for Infectious Disease Research in Zambia<br>Sikombe, Kombatende; Center for Infectious Disease Research in Zambia, Implementation Science Unit, Research department<br>Sikazwe, Izukanji; Center for Infectious Disease Research in Zambia, Chief Executive Officer<br>Bolton-Moore, Carolyn; Center for Infectious Disease Research in Zambia; The University of Alabama at Birmingham School of Medicine, Infectious Disease<br>Mody, Aaloke; Washington University in St Louis, Infectious Diseases<br>Geng, Elvin; Washington University in St Louis School of Medicine<br>Sharma, Anjali; Center for Infectious Disease Research in Zambia<br>Beres, Laura K ; Johns Hopkins University Bloomberg School of Public Health, Department of International Health<br>Pry, Jake; Center for Infectious Disease Research in Zambia, Implementation Science Unit; University of California Davis School of Medicine, Public Health Sciences
**Primary Subject Heading**:	Mental health
Secondary Subject Heading:	HIV/AIDS, Health services research, Haematology (incl blood transfusion), Epidemiology, Global health
Keywords:	COVID-19, EPIDEMIOLOGY, HIV & AIDS < INFECTIOUS DISEASES, MENTAL HEALTH

I, the Submitting Author has the right to grant and does grant on behalf of all authors of the Work (as defined in the below author licence), an exclusive licence and/or a non-exclusive licence for contributions from authors who are: i) UK Crown employees; ii) where BMJ has agreed a CC-BY licence shall apply, and/or iii) in accordance with the terms applicable for US Federal Government officers or employees acting as part of their official duties; on a worldwide, perpetual, irrevocable, royalty-free basis to BMJ Publishing Group Ltd ("BMJ") its licensees and where the relevant Journal is co-owned by BMJ to the co-owners of the Journal, to publish the Work in this journal and any other BMJ products and to exploit all rights, as set out in our licence.

The Submitting Author accepts and understands that any supply made under these terms is made by BMJ to the Submitting Author unless you are acting as an employee on behalf of your employer or a postgraduate student of an affiliated institution which is paying any applicable article publishing charge ("APC") for Open Access articles. Where the Submitting Author wishes to make the Work available on an Open Access basis (and intends to pay the relevant APC), the terms of reuse of such Open Access shall be governed by a Creative Commons licence – details of these licences and which Creative Commons licence will apply to this Work are set out in our licence referred to above.

Other than as permitted in any relevant BMJ Author's Self Archiving Policies, I confirm this Work has not been accepted for publication elsewhere, is not being considered for publication elsewhere and does not duplicate material already published. I confirm all authors consent to publication of this Work and authorise the granting of this licence.

For peer review only

A cross-sectional study to assess depression among health care workers in Lusaka, Zambia during the COVID-19 pandemic.

Sandra Simbeza[1], Jacob Mutale[1], Musunge Mulabe[1], Lazarus Jere[1], Chama Bukankala[1], Kombatende Sikombe[1], Izukanji Sikazwe[1], Carolyn Bolton Moore[1,2], Aaloke Mody[3], Elvin H. Geng[3], Anjali Sharma[1], Laura K. Beres[2†], Jake M. Pry[1,5§†]

[1]Centre for infectious Disease Research in Zambia, Lusaka Zambia
[2]Division of Infectious Diseases, Department of Internal Medicine, School of Medicine, University of Alabama, Birmingham AL, USA
[3] Department of Internal Medicine School of Medicine, Washington University in St. Louis, MO USA
[4]Department of International Health, Johns Hopkins Bloomberg School of Public Health, Baltimore, MD USA
[5]Department of Public Health Sciences, School of Medicine, University of California, Davis, CA, USA

†Authors contributed equally to manuscript

§ Corresponding author: Jake M. Pry

Jake M. Pry, PhD, MPH
Centre for Infectious Disease Research Zambia
Plot 34620 Danny Pule Road, Mass Media
Lusaka, Zambia 10101
jake.pry@cidrz.org | jmpry@ucdavis.edu
Phone: +1 936 661 6885

Keywords: Health Care Workers, Depression, COVID-19, PHQ-9

For peer review only - http://bmjopen.bmj.com/site/about/guidelines.xhtml

Abstract

Objectives: We sought to assess depression among health care workers (HCWs) in the context of COVID-19 in Lusaka Province, Zambia.

Design: This cross-sectional study is nested within a larger study, Person Centered Public Health for HIV Treatment in Zambia (PCPH), a cluster-randomized trial to assess HIV care and outcomes.

Setting: The research was conducted in 24 government-run health facilities during 11 August - 15 October 2020 during the second wave of the COVID-19 epidemic in Lusaka, Zambia.

Participants: We used convenience sampling to recruit HCW participants who were previously enrolled in the PCPH study, had more than 6 months experience working at the facility and were voluntarily willing to participate.

Primary outcome measures: We implemented the well-validated Patient Health Questionnaire (PHQ-9) to assess HCW depression. We used mixed-effects, adjusted Poisson regression to estimate the marginal probability of HCW experiencing depression that may warrant intervention (PHQ-9 score ≥5) by health care facility.

Results: We collected PHQ-9 survey responses from 713 professional and lay HCWs. Overall, 334 (46.8%, 95% CI: 43.1, .50.6%) HCWs recorded a PHQ-9 score ≥5, indicating the need for further assessment and potential intervention for depression We identified significant heterogeneity across facilities and observed a greater proportion of HCWs with symptoms of depression in facilities providing COVID-19 testing and treatment services.

Conclusions: Depression may be a concern for a large proportion of HCW in Zambia. Further work to understand the magnitude and etiologies of depression amongst HCW in the public sector is needed to design effective preventative and treatment interventions to meet the need for mental health support and to minimize poor health outcomes.

For peer review only - http://bmjopen.bmj.com/site/about/guidelines.xhtml

BMJ Open Questions:

Strengths and Limitations of this study

- These data represent important insight regarding the state of mental wellness among a large sample of health care workers during a public health crisis in a resource limited setting.
- Mental wellness, like depression, is not documented well in resource limited settings and we measure here mental wellness in a high-risk group of health care workers during the COVID-19 pandemic.
- We include a large number (N=24) of facilities to appreciate the heterogeneity in symptoms of depression among mental health in the sampled facilities in Lusaka Province of Zambia.
- An important limitation is lack of data on demographics of health care worker respondents due to concern about stigma around mental wellness and potential to identify individuals given inclusion of clinics with small staff.
- Without pre-COVID 19 estimates of depression among health care workers in Lusaka, we are not able to show association between mild-severe depression with the pandemic; however, we show that during the pandemic that depression was high and attention to this population is justified to ensure a healthy HCW workforce.

For peer review only - http://bmjopen.bmj.com/site/about/guidelines.xhtml
For peer review only

Introduction

Coronavirus disease 2019 (COVID-19) has caused substantial global health hardship and health care workers (HCWs) position on the frontlines of the public health response places them at great risk for negative effects on health and wellbeing[1,[1]. In addition to excess occupational hazard of contracting COVID-19, their experience as care givers increases their risk of developing mental health disorders such as anxiety, depression, trauma, insomnia, and stress, which may lead to poor physical and psychological wellbeing [2]. A healthy workforce is critical to effectively managing and mitigating COVID-19, as well as providing continuity of high-quality care for other chronic and acute health conditions [3, 4]. Even prior to COVID-19, countries in sub-Saharan Africa faced limited medical infrastructure, supplies, and an over-burdened workforce, which challenged HCW wellbeing and the provision of quality health care [5, 6]. These challenges, along with pandemic-sensitive barriers such as limited access to personal protective equipment (PPE) further exacerbated HCW stress and vulnerability. While HCWs in resource limited settings have demonstrated resilience, poor mental health is likely to compromise their ability to make decisions, as well as impact patient interactions [7]. Mental health services, like other health resources, are limited, with few trained mental health providers[8]. Understanding mental health and wellbeing among HCWs can catalyze interventions to provide treatment and improve the health care facility environment for the HCWs and patients [8].

Several studies have been conducted to assess mental wellness impact of the pandemic amongst HCWs however, the majority of these studies focus on the continent of Asia and very little data is available for HCWs in Africa [9-11]. Limited data from Kenya and Ethiopia provides evidence that the prevalence of mental disorders such as depression, insomnia and stress were higher among those HCWs caring for patients with COVID-19, or in areas of higher infection prevalence compared to those working with non-COVID-19 patients or less [2, 12-16]. The most prevalent reported mental health conditions among health care workers are depression, insomnia, and anxiety [17]. Characterization of the state of mental wellness during the COVID-19 pandemic in Sub-Saharan African countries among HCWs, specifically in Zambia remains incomplete [12, 18].

As part of a larger patient-centered care study, we collected facility-level measures to understand the context of care at 24 study sites. As a part of establishing care context we assessed mental

For peer review only - http://bmjopen.bmj.com/site/about/guidelines.xhtml

health, specifically depression, using the nine item Patient Health Questionnaire (PHQ-9) in the context of COVID-19 among HCWs in Lusaka Province, Zambia from 11 August 2020 to 15 October 2020 [19].

Methods

Setting

This cross-sectional study of HCW depression is nested within a larger study, Person Centered Public Health for HIV Treatment in Zambia (PCPH), a cluster-randomized trial to assess HIV care and outcomes running from August 2018 to November 2021 across 24 government-funded health facilities in Lusaka Province (Pan African Clinical Trial Registry number: PACTR202101847907585). All PHQ-9 responses were collected between 11 August 2020 and 15 October 2020 among participating facilities that offered HIV care services and varied in size and location. Facilities were assigned codes to protect the identity of the HCW participants.

Study Population

We developed a cohort of HCWs that primarily provide HIV care at one of the 24 health facilities in the PCPH study. This was done by compiling all HCW's contact details from the HCWs who had, at the time of the introduction of the depression study, participated in the HCW survey component of the original PCPH study. The PCPH sample was comprised of both professional HCWs including nurses, pharmacists, midwives, medical doctors, radiographers, and lay HCWS including treatment supporters and general workers. To augment participation in the PHQ-9 data collection, we expanded the PHQ-9 sample to include any HCW willing to participate and had ≥6 months working experience at the study facility. We informed the facility in-charge of the PHQ-9 study opportunity who communicated it to staff members.

Measurements

Trained study research assistants visited health care facilities and discussed the study opportunity in-person with available staff members. Those who expressed interest were screened for eligibility, offered the opportunity to verbally consent and with immediate participation. As a part of the PCPH adaptation to the COVID-19 pandemic, we implemented the nine-question Patient Health Questionnaire (PHQ-9) to screen for presence and severity of depression. The PHQ-9 has been

For peer review only - http://bmjopen.bmj.com/site/about/guidelines.xhtml

previously used in Zambia to screen for likelihood of depression [20], and has been validated in Tanzania and South Africa which are similar settings as Zambia [20, 21]. The PHQ-9 instrument was translated from English into Nyanja and Bemba, the most commonly spoken Zambian languages in Lusaka Province, where the survey was conducted [22]. Depending on the participant preference, the standard PHQ-9 survey was self-administered on paper or surveyor-administered by trained study research assistants in the participant's preferred language. Potentially identifying information such as age, sex, and HCW cadre were not collected from respondents to protect privacy. Facility populations were categorized as small (<40,000 clients/year), medium (40,000-99,999 visits/year), and large (≥100,000 visits/year) as recorded in 2019.

Analysis

We followed the standard, 27-point scoring system for the PHQ-9 to identify participants with scores consistent with minimal (0-4), mild (5-9), moderate (10-14), moderately severe (15-19), and severe depression (20-27) [19]. Our main outcome was mild depression or greater (PHQ-9 score >=5) as this level of depression warrants additional clinical follow-up [19]. We developed frequency tables and used a bar graphs to illustrate the distribution of PHQ-9 scores by health care facility. We developed scatter plots of adjusted marginal probability with 95% confidence intervals to illustrate the probability that a HCW will have a PHQ-9 score ≥5 by health care facility. We used mixed effects Poisson regression to estimate prevalence ratios for those with mild depression allowing random effects at the facility level and measured fixed effects for month of survey and clinic size category. Facilities were categorized by client population estimates as: small (<40,000 clients), medium (40,000-100,000 clients), and large (>100,000 clients).

Patient and Public Involvement

The parent study, focused primarily on improving the patient experience at routine clinic visits through HCW training, mentorship, and audit and feedback The research approach and content is based on participatory research with HCWs, human-centered design workshops, and stakeholder collaborations [23-29]. This research guided us to include measures of HCW satisfaction and wellbeing. In addition to a HCW experience measure throughout PCPH, the advent of COVID-19 led us to include an assessment of HCW depression. Patients were not directly involved in the design, nor the recruitment of this sub-study assessing the depression levels of HCWs. The

For peer review only - http://bmjopen.bmj.com/site/about/guidelines.xhtml

findings of this study as well as the parent study will be shared with the Zambian Ministry of Health as well as the study facilities.

Ethics

This study was approved as part of the larger PCPH study by the University of Zambia Biomedical Ethics Committee and the University of Alabama at Birmingham Institution Review Board, and the National Health Research Authority in Zambia. We obtained waiver of written informed consent and obtained verbal consent from participants prior to administering the survey.

Results

A total of 713 HCWs from 24 facilities across Lusaka and Chongwe districts in the Lusaka Province were included in the analysis dataset (**Table 1**). The majority (69.6%) of the PHQ-9 survey responses were collected in September 2020 (**Table 1**). The largest proportion of the responses were collected at facilities serving a large client population (41.7%) followed closely by facilities serving s medium-sized client population (37.7%) (**Table 1**). Responses for PHQ-9 questions were largely complete with <1% responses missing.

Table 1: Participant characteristics

Factor	Level	n (%)
N		713
Facility	1	30 (4.2)
	2	30 (4.2)
	3	30 (4.2)
	4	30 (4.2)
	5	30 (4.2)
	6	30 (4.2)
	7	30 (4.2)
	8	30 (4.2)
	9	30 (4.2)
	10	30 (4.2)
	11	30 (4.2)
	12	27 (3.8)
	13	27 (3.8)

For peer review only - http://bmjopen.bmj.com/site/about/guidelines.xhtml

For peer review only

	14	30 (4.2)
	15	30 (4.2)
	16	30 (4.2)
	17	30 (4.2)
	18	30 (4.2)
	19	30 (4.2)
	20	30 (4.2)
	21	30 (4.2)
	22	30 (4.2)
	23	29 (4.1)
	24	30 (4.2)
Clinic Population (category)	Small	147 (20.6)
	Medium	269 (37.7)
	Large	297 (41.7)
Month of Survey	August 2020	205 (28.8)
	September 2020	496 (69.6)
	October 2020	12 (1.7)

Note:

Of the 713 responses, 231 (32.4%, 95%CI: 29.0-36.0) reported mild depression (PHQ-9 score 5-9), 81 (11.4% 95%CI: 9.1-13.9) reported moderate depression (PHQ-9 score 10-14). A total of 81 (11.4) respondents had a PHQ-9 score corresponding to moderate depression across all but one facility, 17 (2.4%, 95%CI: 1.4-3.8) respondents had PHQ-9 scores consistent with moderately severe depression across four facilities, and five (0.7%, 95%CI: 0.2-1.6) HCWs had PHQ-9 scores consistent with severe depression across five different facilities (**Fig 1, Fig S1, Table 2**).

Table 2: Proportion of the analysis population by PHQ-9 score category with 95% confidence intervals (N=713)

Variable	*n*	*Proportion (%)*	*95% CI*
≥5 PHQ-9 score	334	46.8	(43.1, 50.6)
Mild	231	32.4	(29.0, 36.0)
Moderate	81	11.4	(9.1, 13.9)
Moderate-severe	17	2.4	(1.4, 3.8)
Severe	5	0.7	(0.2, 1.6)

Note: CI – confidence interval; PHQ-9 – nine-item patient health questionnaire.

For peer review only - http://bmjopen.bmj.com/site/about/guidelines.xhtml

Though we observed facility-level mental health heterogeneity in the proportion of minimal and mild depression scores across clinics, the proportion of scores consistent with moderate depression remain relatively stable across facilities (**Fig 1A).**

Figure 1: A) Stacked bar chart of proportion population by PHQ-9 score category by health care facility B) Stacked bar chart of proportion population by PHQ-9 score by facility population size category

Mixed effects adjusted Poisson regression model did not reveal clinic size or month of survey as a significant predictor of PHQ-9 score ≥5 (**Table 3**).

Table 3: Adjusted Poisson regression results (N=713)

Covariate	*level*	*aPR*	*95% CI*	*p-value*
Clinic Population (category)	small	1.12	(0.95, 1.33)	0.170
	medium	1.00 (ref)	ref	ref
	large	1.04	(0.81, 1.33)	0.763
Month of Survey	August	1.00 (ref)	ref	ref
	September	1.14	(0.94, 1.38)	0.193
	October	0.98	(0.54, 1.79)	0.945

Note: adjusted for survey week and facility

We illustrate significant heterogeneity in the marginal probability of experiencing greater than minimal depression across facilities (**Fig 2**). Notably, the highest marginal probability of HCWs with PHQ-9 score ≥5 was observed in a facility serving as a COVID-19 treatment center.

Figure 2: Marginal probability of health care worker with >minimal depression per PHQ-9 score by clinic.

Note: PHQ-9 – nine-item patient health questionnaire.

Discussion

We found that a large proportion of the HCW population had a PHQ-9 score ≥5 indicating a need for follow-up to assess and improve mental wellbeing 46.8% (95% CI: 43.1, 50.6%). Variation in

For peer review only

depression outcomes ranged from 82.7% (95% CI: 68.5, 96.9%) at one of the two large COVID-19 treatment facilities to 28.4% (95% CI: 13.0, 43.9%) at a medium-sized health care facility.

Our study shows that HCWs working at COVID-19 treatment facilities had a higher marginal probability of experiencing mild to moderately severe depression. This is consistent with past studies showing that frontline HCW working in clinics managing diseases considered to be highly infectious, such as COVID-19 treatment centers, were more prone to developing depression and other mental disorders than their counterparts in other departments [12]. In addition, results of our study demonstrate that HCWs have experienced symptoms of depression during the onset of the COVID-19 pandemic in Zambia, which is consistent with the findings of similar studies using the PHQ-9 in other parts of the world where a pooled prevalence of mild depression was found to be 36.1% (95% confidence interval [CI], 31.3%-41.0%) [30]. Studies conducted in Zambia among nurses and midwives, and in Ethiopia among different cadres of HCWs show that mental disorders which include depression are prevalent among HCWs during the COVID-19 pandemic [31]. Similarly, globally studies indicate that there is a risk of HCWs developing mental health disorders during the pandemic [4, 30, 31]. The difficult conditions to which HCWs may be exposed including extended working hours, risk of exposure to the disease, increased workload, concerns about transmitting the infection to their family members, reduced social connectedness, and limited resources to care for their patients may amplify poor mental wellbeing [1, 32-34].

Further research is needed to understand heterogeneity in proportions of HCWs with depression. It may be associated variation in HCW access to resources such as, personal protective equipment (PPE), hand hygiene station/station supplies at clinic entrance, and knowledge about the COVID-19 response in Zambia. While the response to the COVID-19 pandemic was standardized, to a certain extent, by guidance from the provincial and zonal levels, the culture and leadership unique to each facility might have played a key role in the health care worker experience, contributing to PHQ-9 score distribution differences across facilities.

As we continue to recognize the mental health services gap across many populations in resource-limited settings we give evidence here to support prioritization of health care workers, especially during public health shocks/emergencies like that presented by COVID-19.Presenceof depression

For peer review only - http://bmjopen.bmj.com/site/about/guidelines.xhtml

among HCWs could lead to poor health outcomes for the HCW workforce and have a sort of "knock-on" effect negatively impacting the quality of care provided to patients [35, 36]. Interventions like Friendship Bench piloted in neighboring Zimbabwe designed to encourage positive coping mechanism among HCWs and build a working environment that provides empathy and compassion towards staff may be an efficient option to provide mental wellness support [37, 38]. Furthermore, system-based interventions should also be encouraged such as change in working culture and reduction in possible system contributors to HCW stress that could lead to depression. Increasing mobile technology availability may further allow for the use of mobile health (mHealth) based mental wellness services leveraging the framework presented by Osei for low- and middle-income countries [39, 40]. Low-cost intervention packages used for patients can be adapted for HCWs and integrated into system-based interventions. They include routine screening for early detection, mental wellness education, problem solving and use of anti-depressants, cognitive behavior therapy which can be delivered successfully by trained lay HCWs [41, 42].

Limitations

This study had some potential limitations. Firstly, we did not collect participants demographics such as age, sex, marital status, and sex. As such we were not able to adjust for these co- founders. Accounting for these would help make associations in outcomes of depression. Secondly, we employed convenience sampling in selecting participants, this sampling approach may limit representativeness of study the results to the wider population of HCWs. Finally, without pre-COVID 19 estimates of depression among health care workers in Lusaka, we are not able to show association between mild-severe depression with the pandemic however, we show that during the pandemic that depression was high and attention to this population is justified to ensure a healthy HCW workforce. Additionally, though potentially highest than a non-pandemic baseline, these results may be useful as subsequent measures of depression and mental wellness are collected among HCWs.

Conclusions

Depression is a common public health problem; our study demonstrates that HCWs in Zambia may suffer from a high prevalence of depressive symptoms that will require additional clinical

For peer review only - http://bmjopen.bmj.com/site/about/guidelines.xhtml

follow up. Routine mental health wellness is important to better understand the role that the COVID-19 pandemic may have had on depression among HCWS in Zambia. Furthermore, support for HCW mental wellness can serve to accelerate destigmatizing mental health issues and improve the quality of care provided across health care centers in Zambia.

For peer review only

For peer review only - http://bmjopen.bmj.com/site/about/guidelines.xhtml

Contributorship

SS: data collection, data Framing, and manuscript writing, JM, KS, and MM: data collection and data curation, LJ and CB: data framing, IS, CBM and EHG: conceptualization, AM: manuscript review/editing, AS: conceptualization and manuscript review/editing LKB: conceptualization, data analysis, data framing, and manuscript writing, JMP: data analysis lead, data framing, and manuscript writing. The corresponding author attests that all listed authors meet authorship criteria and that no others meeting the criteria have been omitted.

Funding

Research reported in this publication was supported by the Bill & Melinda Gates Foundation grant number OPP1166485, the National Institute of Mental Health of the National Institutes of Health under award number F31MH109378 (L.K.B.), and the National Institute of Allergy and Infectious Diseases of the National Institutes of Health under award number K24 AI134413 (E.G.).

Competing of Interests

None

Ethical Approval

This study was approved as part of the larger PCPH study by the University of Zambia Biomedical Ethics Committee and the University of Alabama at Birmingham Institution Review Board, and the National Health Research Authority in Zambia. We obtained waiver of written informed consent and obtained verbal consent from participants prior to administering the survey.

Data Sharing

Data used for all analyses will be made available for public use through Dryad data repository: Pry, Jake et al. (2022), PHQ-9_PCPH-19aug2022, https://doi.org/10.25338/B8S646.

Acknowledgements

We would like to thank the Zambian Ministry of Health for their leadership to ensure those in care continue to receive life-saving HIV treatment. We would also like to thank the health care

For peer review only - http://bmjopen.bmj.com/site/about/guidelines.xhtml

workers for their participation in the PHQ-9 assessment as well as their faithful delivery of care to patients in the face of the many challenges presented by the COVID-19 pandemic. To the clients seen at our study facilities we would like to extend sincere thanks for your cooperation and patience.

List of abbreviations [Optional]

ART: antiretroviral therapy
CI- Confidence Interval
COVID-19: Coronavirus Disease 2019
HCW: Health Care Worker
PCPH: Person- Centered Public Health for HIV Treatment in Zambia
PHQ-9: Patient Health Questionnaire 9

Data Sharing

Data used for this analysis is available through Dryad data repository (doi:10.25338/B8S646)

References

1. Yıldırım M, Çiçek İ, Şanlı ME. Coronavirus stress and COVID-19 burnout among healthcare staffs: The mediating role of optimism and social connectedness. Curr Psychol. 2021;40(11):5763-71. Epub 2021/05/04. doi: 10.1007/s12144-021-01781-w. PubMed PMID: 33935470; PubMed Central PMCID: PMCPMC8064417.
2. Robertson L, Maposa I, Somaroo H, Johnson O. Mental health of healthcare workers during the COVID-19 outbreak: A rapid scoping review to inform provincial guidelines in South Africa. South African Medical Journal. 2020;110(10):1010-9.
3. Gupta S, Sahoo S. Pandemic and mental health of the front-line healthcare workers: a review and implications in the Indian context amidst COVID-19. General Psychiatry. 2020;33(5).
4. Saragih ID, Tonapa SI, Saragih IS, Advani S, Batubara SO, Suarilah I, et al. Global Prevalence of Mental Health Problems among healthcare workers during The Covid-19 Pandemic: A Systematic Review and Meta-Analysis. International Journal of Nursing Studies. 2021:104002.
5. Ayanore MA, Amuna N, Aviisah M, Awolu A, Kipo-Sunyehzi DD, Mogre V, et al. Towards resilient health systems in sub-saharan africa: A systematic review of the english language literature on health workforce, surveillance, and health governance issues for health systems strengthening. Annals of global health. 2019;85(1).
6. Semo B-w, Frissa SM. The mental health impact of the COVID-19 pandemic: implications for sub-Saharan Africa. Psychology Research and Behavior Management. 2020;13:713.

For peer review only

For peer review only - http://bmjopen.bmj.com/site/about/guidelines.xhtml

7. Med S. Serious Health Workforce Shortage in Zambia: Solidar Med; 2020 [updated 22 December 2020; cited 2022 16 January 2022]. Available from: https://www.solidarmed.ch/en/news/shortage-skilled-workers-health-system-zambia.

8. Munakampe MN. Strengthening mental health systems in Zambia. International journal of mental health systems. 2020;14(1):1-9.

9. Deng D, Naslund JA. Psychological impact of COVID-19 Pandemic on frontline health workers in low-and middle-income countries. Harvard public health review (Cambridge, Mass). 2020;28.

10. Moitra M, Rahman M, Collins PY, Gohar F, Weaver M, Kinuthia J, et al. Mental health consequences for healthcare workers during the COVID-19 pandemic: a scoping review to draw lessons for LMICs. Frontiers in psychiatry. 2021;12:22.

11. Ceri V, Cicek I. Psychological Well-Being, Depression and Stress During COVID-19 Pandemic in Turkey: A Comparative Study of Healthcare Professionals and Non-Healthcare Professionals. Psychology, health & medicine. 2021;26(1):85-97. Epub 2020/12/16. doi: 10.1080/13548506.2020.1859566. PubMed PMID: 33320723.

12. Shah J, Monroe-Wise A, Talib Z, Nabiswa A, Said M, Abeid A, et al. Mental health disorders among healthcare workers during the COVID-19 pandemic: a cross-sectional survey from three major hospitals in Kenya. BMJ open. 2021;11(6):e050316.

13. Chersich MF, Gray G, Fairlie L, Eichbaum Q, Mayhew S, Allwood B, et al. COVID-19 in Africa: care and protection for frontline healthcare workers. Global Health. 2020;16(1):46. Epub 2020/05/18. doi: 10.1186/s12992-020-00574-3. PubMed PMID: 32414379; PubMed Central PMCID: PMCPMC7227172.

14. Yitayih Y, Mekonen S, Zeynudin A, Mengistie E, Ambelu A. Mental health of healthcare professionals during the early stage of the COVID-19 pandemic in Ethiopia. BJPsych Open. 2020;7(1):e1. Epub 2020/12/02. doi: 10.1192/bjo.2020.130. PubMed PMID: 33256883; PubMed Central PMCID: PMCPMC7844150.

15. Jemal K, Deriba BS, Geleta TA, Tesema M, Awol M, Mengistu E, et al. Self-Reported Symptoms of Depression, Anxiety, and Stress Among Healthcare Workers in Ethiopia During the COVID-19 Pandemic: A Cross-Sectional Study. Neuropsychiatr Dis Treat. 2021;17:1363-73. Epub 2021/05/15. doi: 10.2147/NDT.S306240. PubMed PMID: 33986596; PubMed Central PMCID: PMCPMC8110274.

16. Asnakew S, Amha H, Kassew T. Mental Health Adverse Effects of COVID-19 Pandemic on Health Care Workers in North West Ethiopia: A Multicenter Cross-Sectional Study. Neuropsychiatr Dis Treat. 2021;17:1375-84. Epub 2021/05/18. doi: 10.2147/NDT.S306300. PubMed PMID: 33994787; PubMed Central PMCID: PMCPMC8113010.

17. Sahebi A, Nejati-Zarnaqi B, Moayedi S, Yousefi K, Torres M, Golitaleb M. The prevalence of anxiety and depression among healthcare workers during the COVID-19 pandemic: An umbrella review of meta-analyses. Prog Neuropsychopharmacol Biol Psychiatry. 2021;107:110247. Epub 2021/01/22. doi: 10.1016/j.pnpbp.2021.110247. PubMed PMID: 33476692; PubMed Central PMCID: PMCPMC7817526.

18. Elbay RY, Kurtulmuş A, Arpacıoğlu S, Karadere E. Depression, anxiety, stress levels of physicians and associated factors in Covid-19 pandemics. Psychiatry research. 2020;290:113130.

19. Kroenke K, Spitzer RL, Williams JB. The PHQ-9: validity of a brief depression severity measure. Journal of general internal medicine. 2001;16(9):606-13. doi: 10.1046/j.1525-1497.2001.016009606.x. PubMed PMID: 11556941.

For peer review only - http://bmjopen.bmj.com/site/about/guidelines.xhtml

20. Kroenke K, Spitzer RL, Williams JB. The PHQ-9: validity of a brief depression severity measure. Journal of general internal medicine. 2001;16(9):606-13.

21. Kroenke K, Spitzer RL, Williams JB, Löwe B. The patient health questionnaire somatic, anxiety, and depressive symptom scales: a systematic review. General hospital psychiatry. 2010;32(4):345-59.

22. Sawka KS, Mbewe C, Josephat D, Ngulube E. The Senga of Zambia: Linguistic Survey Report with Recommendations for Literature Development Strategy. SIL International: 2021.

23. Beres LK, Simbeza S, Holmes CB, Mwamba C, Mukamba N, Sharma A, et al. Human-centered design lessons for implementation science: improving the implementation of a patient-centered care intervention. Journal of acquired immune deficiency syndromes (1999). 2019;82(3):S230.

24. Mody A, Tram KH, Glidden DV, Eshun-Wilson I, Sikombe K, Mehrotra M, et al. Novel Longitudinal Methods for Assessing Retention in Care: a Synthetic Review. Current HIV/AIDS Reports. 2021;18(4):299-308.

25. Sikombe K, Mody A, Kadota J, Pry JJ, Simbeza S, Eshun-Wilson I, et al. Understanding patient transfers across multiple clinics in Zambia among HIV infected adults. PloS one. 2020;15(11):e0241477.

26. Holmes CB, Sanne I. Changing models of care to improve progression through the HIV treatment cascade in different populations. Current Opinion in HIV and AIDS. 2015;10(6):447-50.

27. Holmes CB, Sikazwe I, Sikombe K, Eshun-Wilson I, Czaicki N, Beres LK, et al. Estimated mortality on HIV treatment among active patients and patients lost to follow-up in 4 provinces of Zambia: Findings from a multistage sampling-based survey. PLoS Med. 2018;15(1):e1002489. Epub 2018/01/13. doi: 10.1371/journal.pmed.1002489. PubMed PMID: 29329301; PubMed Central PMCID: PMCPMC5766235.

28. Roy M, Czaicki N, Holmes C, Chavan S, Tsitsi A, Odeny T, et al. Understanding Sustained Retention in HIV/AIDS Care and Treatment: a Synthetic Review. Current HIV/AIDS Reports. 2016:1-9.

29. Mwamba C, Sharma A, Mukamba N, Beres L, Geng E, Holmes CB, et al. 'They care rudely!': resourcing and relational health system factors that influence retention in care for people living with HIV in Zambia. BMJ Glob Health. 2018;3(5):e001007. Epub 2018/11/30. doi: 10.1136/bmjgh-2018-001007. PubMed PMID: 30483408; PubMed Central PMCID: PMCPMC6231098.

30. Li Y, Scherer N, Felix L, Kuper H. Prevalence of depression, anxiety and post-traumatic stress disorder in health care workers during the COVID-19 pandemic: A systematic review and meta-analysis. PloS one. 2021;16(3):e0246454.

31. Mulatu HA, Tesfaye M, Woldeyes E, Bayisa T, Fisseha H, Kassu RA. The prevalence of common mental disorders among healthcare professionals during the COVID-19 pandemic at a tertiary Hospital in Addis Ababa, Ethiopia. Journal of Affective Disorders Reports. 2021;6:100246. doi: https://doi.org/10.1016/j.jadr.2021.100246.

32. Mokhtari R, Moayedi S, Golitaleb M. COVID-19 pandemic and health anxiety among nurses of intensive care units. International journal of mental health nursing. 2020;29(6):1275.

33. Pfefferbaum B, North CS. Mental health and the Covid-19 pandemic. New England Journal of Medicine. 2020;383(6):510-2.

34. Yıldırım M, Çiçek İ. Optimism and pessimism mediate the association between parental coronavirus anxiety and depression among healthcare professionals in the era of COVID-19.

For peer review only

For peer review only - http://bmjopen.bmj.com/site/about/guidelines.xhtml

Psychology, health & medicine. 2022;27(9):1898-906. Epub 2021/08/13. doi: 10.1080/13548506.2021.1966702. PubMed PMID: 34382894.
35. Saasa Y. An Assessment on the Role of Human Rights and Governance in Health Service Delivery 2019.
36. Kola L, Kohrt BA, Hanlon C, Naslund JA, Sikander S, Balaji M, et al. COVID-19 mental health impact and responses in low-income and middle-income countries: reimagining global mental health. The Lancet Psychiatry. 2021;8(6):535-50. doi: https://doi.org/10.1016/S2215-0366(21)00025-0.
37. Abraham A, Chaabna K, Doraiswamy S, Bhagat S, Sheikh J, Mamtani R, et al. Depression among healthcare workers in the Eastern Mediterranean Region: a systematic review and meta-analysis. Human resources for health. 2021;19(1):1-18.
38. Wallén A, Eberhard S, Landgren K. The Experiences of Counsellors Offering Problem-Solving Therapy for Common Mental Health Issues at the Youth Friendship Bench in Zimbabwe. Issues in Mental Health Nursing. 2021;42(9):808-17. doi: 10.1080/01612840.2021.1879977.
39. Osei E, Mashamba-Thompson TP. Mobile health applications for disease screening and treatment support in low-and middle-income countries: A narrative review. Heliyon. 2021;7(3):e06639. Epub 2021/04/20. doi: 10.1016/j.heliyon.2021.e06639. PubMed PMID: 33869857; PubMed Central PMCID: PMCPMC8035664.
40. Mutimushi PM, Mupenda BM, Manda PM, Nankonde N, Banda B, Mung'omba K, et al. ZICTA 2019 Annual Report. https://www.zicta.zm/storage/posts/attachments/oiBT4oMnpJoN3dgHKpvByoDmNXq5ZDYtp3sxVYM3.pdf: Zambia Information and Communications Technology Authority (ZICTA), 2019.
41. Chibanda D, Mesu P, Kajawu L, Cowan F, Araya R, Abas MA. Problem-solving therapy for depression and common mental disorders in Zimbabwe: piloting a task-shifting primary mental health care intervention in a population with a high prevalence of people living with HIV. BMC public health. 2011;11(1):1-10.
42. Patel V, Simon G, Chowdhary N, Kaaya S, Araya R. Packages of care for depression in low-and middle-income countries. PLoS medicine. 2009;6(10):e1000159.

Supplement:

Figure S1: Histogram of total PHQ-9 scores among study participants

For peer review only - http://bmjopen.bmj.com/site/about/guidelines.xhtml

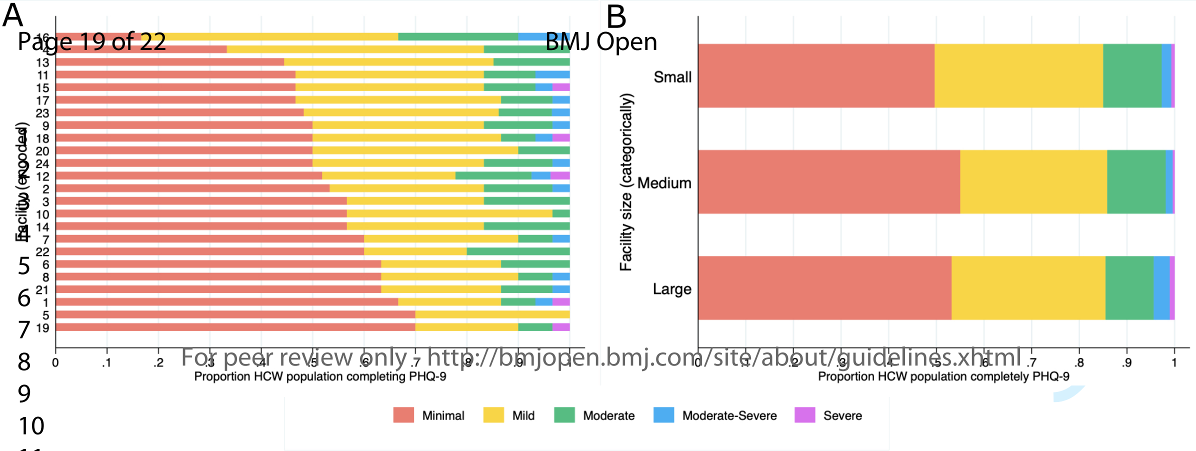
A
16
4
13
11
15
17
23
9
18
20
24
12
2
3
10
14
7
22
6
8
21
1
5
19
Facility (encoded)
0 .1 .2 .3 .4 .5 .6 .7 .8 .9 1
Proportion HCW population completing PHQ-9
B
Small
Medium
Large
Facility size (categorically)
0 .1 .2 .3 .4 .5 .6 .7 .8 .9 1
Proportion HCW population completely PHQ-9
Minimal
Mild
Moderate
Moderate-Severe
Severe

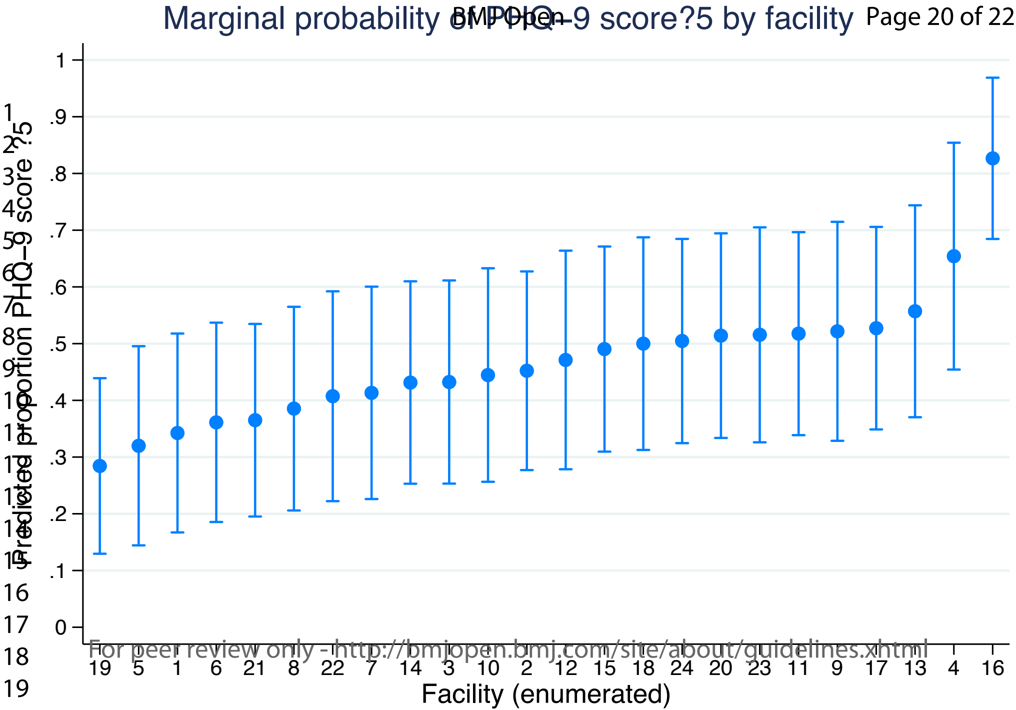

For peer review only - http://bmjopen.bmj.com/site/about/guidelines.xhtml

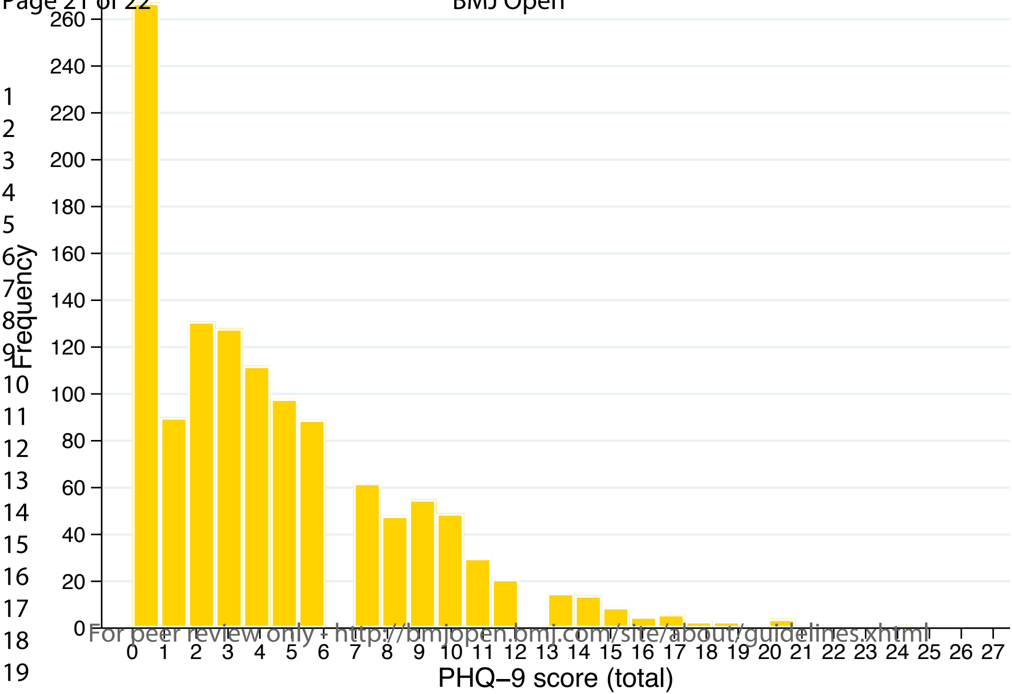

For peer review only - http://bmjopen.bmj.com/site/about/guidelines.xhtml

STROBE Statement—Checklist of items that should be included in reports of ***cohort studies***

	Item No	Recommendation	Page No
**Title and abstract**	1	(*a*) Indicate the study's design with a commonly used term in the title or the abstract	2
		(*b*) Provide in the abstract an informative and balanced summary of what was done and what was found	2
**Introduction**			
Background/rationale	2	Explain the scientific background and rationale for the investigation being reported	4
Objectives	3	State specific objectives, including any prespecified hypotheses	4, 5
**Methods**			
Study design	4	Present key elements of study design early in the paper	5
Setting	5	Describe the setting, locations, and relevant dates, including periods of recruitment, exposure, follow-up, and data collection	5
Participants	6	(*a*) Give the eligibility criteria, and the sources and methods of selection of participants. Describe methods of follow-up	5, 6
		(*b*) For matched studies, give matching criteria and number of exposed and unexposed	NA
Variables	7	Clearly define all outcomes, exposures, predictors, potential confounders, and effect modifiers. Give diagnostic criteria, if applicable	5, 6
Data sources/ measurement	8*	For each variable of interest, give sources of data and details of methods of assessment (measurement). Describe comparability of assessment methods if there is more than one group	5, 6
Bias	9	Describe any efforts to address potential sources of bias	5, 6
Study size	10	Explain how the study size was arrived at	5
Quantitative variables	11	Explain how quantitative variables were handled in the analyses. If applicable, describe which groupings were chosen and why	5, 6
Statistical methods	12	(*a*) Describe all statistical methods, including those used to control for confounding	6
		(*b*) Describe any methods used to examine subgroups and interactions	6
		(*c*) Explain how missing data were addressed	6
		(*d*) If applicable, explain how loss to follow-up was addressed	NA
		(*e*) Describe any sensitivity analyses	6
**Results**			
Participants	13*	(a) Report numbers of individuals at each stage of study—eg numbers potentially eligible, examined for eligibility, confirmed eligible, included in the study, completing follow-up, and analysed	7, 8
		(b) Give reasons for non-participation at each stage	NA
		(c) Consider use of a flow diagram	NA
Descriptive data	14*	(a) Give characteristics of study participants (eg demographic, clinical, social) and information on exposures and potential confounders	7, 8
		(b) Indicate number of participants with missing data for each variable of interest	7, 8
		(c) Summarise follow-up time (eg, average and total amount)	NA
Outcome data	15*	Report numbers of outcome events or summary measures over time	7, 8

For peer review only

Main results	16	(*a*) Give unadjusted estimates and, if applicable, confounder-adjusted estimates and their precision (eg, 95% confidence interval). Make clear which confounders were adjusted for and why they were included	7, 8
		(*b*) Report category boundaries when continuous variables were categorized	7, 8
		(*c*) If relevant, consider translating estimates of relative risk into absolute risk for a meaningful time period	NA
Other analyses	17	Report other analyses done—eg analyses of subgroups and interactions, and sensitivity analyses	9
**Discussion**			
Key results	18	Summarise key results with reference to study objectives	10
Limitations	19	Discuss limitations of the study, taking into account sources of potential bias or imprecision. Discuss both direction and magnitude of any potential bias	11, 12
Interpretation	20	Give a cautious overall interpretation of results considering objectives, limitations, multiplicity of analyses, results from similar studies, and other relevant evidence	9, 10
Generalisability	21	Discuss the generalisability (external validity) of the study results	9, 10
**Other information**			
Funding	22	Give the source of funding and the role of the funders for the present study and, if applicable, for the original study on which the present article is based	13, 14

*Give information separately for exposed and unexposed groups.

Note: An Explanation and Elaboration article discusses each checklist item and gives methodological background and published examples of transparent reporting. The STROBE checklist is best used in conjunction with this article (freely available on the Web sites of PLoS Medicine at http://www.plosmedicine.org/, Annals of Internal Medicine at http://www.annals.org/, and Epidemiology at http://www.epidem.com/). Information on the STROBE Initiative is available at http://www.strobe-statement.org.

For peer review only

A cross-sectional study to assess depression among health care workers in Lusaka, Zambia during the COVID-19 pandemic

Journal:	*BMJ Open*
Manuscript ID	bmjopen-2022-069257.R2
Article Type:	Original research
Date Submitted by the Author:	08-Mar-2023
Complete List of Authors:	Simbeza, Sandra; Center for Infectious Disease Research in Zambia<br>Mutale, Jacob; Center for Infectious Disease Research in Zambia, Implementation Science Unit<br>Mulabe, Musunge; Center for Infectious Disease Research in Zambia<br>Jere, Lazarus; Center for Infectious Disease Research in Zambia<br>Bukankala, Chama; Center for Infectious Disease Research in Zambia<br>Sikombe, Kombatende; Center for Infectious Disease Research in Zambia, Implementation Science Unit, Research department<br>Sikazwe, Izukanji; Center for Infectious Disease Research in Zambia, Chief Executive Officer<br>Bolton-Moore, Carolyn; Center for Infectious Disease Research in Zambia; The University of Alabama at Birmingham School of Medicine, Infectious Disease<br>Mody, Aaloke; Washington University in St Louis, Infectious Diseases<br>Geng, Elvin; Washington University in St Louis School of Medicine<br>Sharma, Anjali; Center for Infectious Disease Research in Zambia<br>Beres, Laura K ; Johns Hopkins University Bloomberg School of Public Health, Department of International Health<br>Pry, Jake; Center for Infectious Disease Research in Zambia, Implementation Science Unit; University of California Davis School of Medicine, Public Health Sciences
**Primary Subject Heading**:	Mental health
Secondary Subject Heading:	HIV/AIDS, Health services research, Epidemiology, Global health
Keywords:	COVID-19, EPIDEMIOLOGY, HIV & AIDS < INFECTIOUS DISEASES, MENTAL HEALTH

SCHOLARONE™ Manuscripts

I, the Submitting Author has the right to grant and does grant on behalf of all authors of the Work (as defined in the below author licence), an exclusive licence and/or a non-exclusive licence for contributions from authors who are: i) UK Crown employees; ii) where BMJ has agreed a CC-BY licence shall apply, and/or iii) in accordance with the terms applicable for US Federal Government officers or employees acting as part of their official duties; on a worldwide, perpetual, irrevocable, royalty-free basis to BMJ Publishing Group Ltd ("BMJ") its licensees and where the relevant Journal is co-owned by BMJ to the co-owners of the Journal, to publish the Work in this journal and any other BMJ products and to exploit all rights, as set out in our licence.

The Submitting Author accepts and understands that any supply made under these terms is made by BMJ to the Submitting Author unless you are acting as an employee on behalf of your employer or a postgraduate student of an affiliated institution which is paying any applicable article publishing charge ("APC") for Open Access articles. Where the Submitting Author wishes to make the Work available on an Open Access basis (and intends to pay the relevant APC), the terms of reuse of such Open Access shall be governed by a Creative Commons licence – details of these licences and which Creative Commons licence will apply to this Work are set out in our licence referred to above.

Other than as permitted in any relevant BMJ Author's Self Archiving Policies, I confirm this Work has not been accepted for publication elsewhere, is not being considered for publication elsewhere and does not duplicate material already published. I confirm all authors consent to publication of this Work and authorise the granting of this licence.

For peer review only

A cross-sectional study to assess depression among health care workers in Lusaka, Zambia during the COVID-19 pandemic.

Sandra Simbeza[1], Jacob Mutale[1], Musunge Mulabe[1], Lazarus Jere[1], Chama Bukankala[1], Kombatende Sikombe[1], Izukanji Sikazwe[1], Carolyn Bolton Moore[1,2], Aaloke Mody[3], Elvin H. Geng[3], Anjali Sharma[1], Laura K. Beres[2†], Jake M. Pry[1,5§†]

[1]Centre for infectious Disease Research in Zambia, Lusaka Zambia
[2]Division of Infectious Diseases, Department of Internal Medicine, School of Medicine, University of Alabama, Birmingham AL, USA
[3] Department of Internal Medicine School of Medicine, Washington University in St. Louis, MO USA
[4]Department of International Health, Johns Hopkins Bloomberg School of Public Health, Baltimore, MD USA
[5]Department of Public Health Sciences, School of Medicine, University of California, Davis, CA, USA

[†]Authors contributed equally to manuscript

[§] Corresponding author: Jake M. Pry

Jake M. Pry, PhD, MPH
Centre for Infectious Disease Research Zambia
Plot 34620 Danny Pule Road, Mass Media
Lusaka, Zambia 10101
jake.pry@cidrz.org | jmpry@ucdavis.edu
Phone: +1 936 661 6885

Keywords: Health Care Workers, Depression, COVID-19, PHQ-9

For peer review only - http://bmjopen.bmj.com/site/about/guidelines.xhtml

Abstract

Objectives: We sought to assess depression among health care workers (HCWs) in the context of COVID-19 in Lusaka Province, Zambia.

Design: This cross-sectional study is nested within a larger study, Person Centered Public Health for HIV Treatment in Zambia (PCPH), a cluster-randomized trial to assess HIV care and outcomes.

Setting: The research was conducted in 24 government-run health facilities during 11 August - 15 October 2020 during the first wave of the COVID-19 epidemic in Lusaka, Zambia.

Participants: We used convenience sampling to recruit HCW participants who were previously enrolled in the PCPH study, had more than 6 months experience working at the facility and were voluntarily willing to participate.

Primary outcome measures: We implemented the well-validated Patient Health Questionnaire (PHQ-9) to assess HCW depression. We used mixed-effects, adjusted Poisson regression to estimate the marginal probability of HCW experiencing depression that may warrant intervention (PHQ-9 score ≥5) by health care facility.

Results: We collected PHQ-9 survey responses from 713 professional and lay HCWs. Overall, 334 (46.8%, 95% CI: 43.1, .50.6%) HCWs recorded a PHQ-9 score ≥5, indicating the need for further assessment and potential intervention for depression We identified significant heterogeneity across facilities and observed a greater proportion of HCWs with symptoms of depression in facilities providing COVID-19 testing and treatment services.

Conclusions: Depression may be a concern for a large proportion of HCW in Zambia. Further work to understand the magnitude and etiologies of depression amongst HCW in the public sector is needed to design effective preventative and treatment interventions to meet the need for mental health support and to minimize poor health outcomes.

For peer review only - http://bmjopen.bmj.com/site/about/guidelines.xhtml

BMJ Open Questions:

Strengths and Limitations of this study

- These data represent important insights regarding the state of mental wellness among a large sample of health care workers during a public health crisis in a resource limited setting, where mental wellness, such as depression, is often not measured nor documented.
- We include a large number (N=24) of facilities to appreciate the heterogeneity in symptoms of depression among mental health in the sampled facilities in Lusaka Province of Zambia.
- An important limitation is lack of data on demographics of health care worker respondents due to concern about stigma around mental wellness and potential to identify individuals given inclusion of clinics with small staff.
- Without pre-COVID 19 estimates of depression among health care workers in Lusaka, we are not able to show association between mild-severe depression with the pandemic; however, we show that during the pandemic that depression was high and attention to this population is justified to ensure a healthy HCW workforce.
- We employed convenience sampling in selecting participants which may limit representativeness of study results to the wider population of HCWs.

For peer review only

Introduction

Coronavirus disease 2019 (COVID-19) has caused substantial global health hardship and health care workers (HCWs) position on the frontlines of the public health response places them at great risk for negative effects on health and wellbeing[1,[1]. In addition to excess occupational hazard of contracting COVID-19, their experience as care givers increases their risk of developing mental health disorders such as anxiety, depression, trauma, insomnia, and stress, which may lead to poor physical and psychological wellbeing [2]. A healthy workforce is critical to effectively managing and mitigating COVID-19, as well as providing continuity of high-quality care for other chronic and acute health conditions [3, 4]. Even prior to COVID-19, countries in sub-Saharan Africa faced limited medical infrastructure, supplies, and an over-burdened workforce, which challenged HCW wellbeing and the provision of quality health care [5, 6]. These challenges, along with pandemic-sensitive barriers such as limited access to personal protective equipment (PPE) further exacerbated HCW stress and vulnerability. While HCWs in resource limited settings have demonstrated resilience, poor mental health is likely to compromise their ability to make decisions, as well as impact patient interactions [7]. Mental health services, like other health resources, are limited, with few trained mental health providers[8]. Understanding mental health and wellbeing among HCWs can catalyze interventions to provide treatment and improve the health care facility environment for the HCWs and patients [8].

Several studies have been conducted to assess mental wellness impact of the pandemic amongst HCWs however, the majority of these studies focus on the continent of Asia and very little data is available for HCWs in Africa [9-11]. Limited data from Kenya and Ethiopia provides evidence that the prevalence of mental disorders such as depression, insomnia and stress were higher among those HCWs caring for patients with COVID-19, or in areas of higher infection prevalence compared to those working with non-COVID-19 patients or less [2, 12-16]. The most prevalent reported mental health conditions among health care workers are depression, insomnia, and anxiety [17]. Characterization of the state of mental wellness during the COVID-19 pandemic in Sub-Saharan African countries among HCWs, specifically in Zambia remains incomplete [12, 18].

As part of a larger patient-centered care study, we collected facility-level measures to understand the context of care at 24 study sites. As a part of establishing care context we assessed mental

For peer review only - http://bmjopen.bmj.com/site/about/guidelines.xhtml

health, specifically depression, using the nine item Patient Health Questionnaire (PHQ-9) in the context of COVID-19 among HCWs in Lusaka Province, Zambia from 11 August 2020 to 15 October 2020 [19].

Methods

Setting

This cross-sectional study of HCW depression is nested within a larger study, Person Centered Public Health for HIV Treatment in Zambia (PCPH), a cluster-randomized trial to assess HIV care and outcomes running from August 2018 to November 2021 across 24 government-funded health facilities in Lusaka Province (Pan African Clinical Trial Registry number: PACTR202101847907585). All PHQ-9 responses were collected between 11 August 2020 and 15 October 2020 among participating facilities that offered HIV care services and varied in size and location. Facilities were assigned codes to protect the identity of the HCW participants.

Study Population

We developed a cohort of HCWs that primarily provide HIV care at one of the 24 health facilities in the PCPH study. This was done by compiling all HCW's contact details from the HCWs who had, at the time of the introduction of the depression study, participated in the HCW survey component of the original PCPH study. The PCPH sample was comprised of both professional HCWs including nurses, pharmacists, midwives, medical doctors, radiographers, and lay HCWS including treatment supporters and general workers. To augment participation in the PHQ-9 data collection, we expanded the PHQ-9 sample to include any HCW willing to participate and had ≥6 months working experience at the study facility. We informed the facility in-charge of the PHQ-9 study opportunity who communicated it to staff members.

Measurements

Trained study research assistants visited health care facilities and discussed the study opportunity in-person with available staff members. Those who expressed interest were screened for eligibility, offered the opportunity to verbally consent and with immediate participation. As a part of the PCPH adaptation to the COVID-19 pandemic, we implemented the nine-question Patient Health Questionnaire (PHQ-9) to screen for presence and severity of depression. The PHQ-9 has been

For peer review only - http://bmjopen.bmj.com/site/about/guidelines.xhtml

For peer review only

previously used in Zambia to screen for likelihood of depression [20], and has been validated in Tanzania and South Africa which are similar settings as Zambia [20, 21]. The PHQ-9 instrument was translated from English into Nyanja and Bemba, the most commonly spoken Zambian languages in Lusaka Province, where the survey was conducted [22]. Depending on the participant preference, the standard PHQ-9 survey was self-administered on paper or surveyor-administered by trained study research assistants in the participant's preferred language. Potentially identifying information such as age, sex, and HCW cadre were not collected from respondents to protect privacy. Facility populations were categorized as small (<40,000 clients/year), medium (40,000-99,999 visits/year), and large (≥100,000 visits/year) as recorded in 2019.

Analysis

We followed the standard, 27-point scoring system for the PHQ-9 to identify participants with scores consistent with minimal (0-4), mild (5-9), moderate (10-14), moderately severe (15-19), and severe depression (20-27) [19]. Our main outcome was mild depression or greater (PHQ-9 score >=5) as this level of depression warrants additional clinical follow-up [19]. We developed frequency tables and used a bar graphs to illustrate the distribution of PHQ-9 scores by health care facility. We developed scatter plots of adjusted marginal probability with 95% confidence intervals to illustrate the probability that a HCW will have a PHQ-9 score ≥5 by health care facility. We used mixed effects Poisson regression to estimate prevalence ratios for those with mild depression allowing random effects at the facility level and measured fixed effects for month of survey and clinic size category. Facilities were categorized by client population estimates as: small (<40,000 clients), medium (40,000-100,000 clients), and large (>100,000 clients).

Patient and Public Involvement

The parent study, focused primarily on improving the patient experience at routine clinic visits through HCW training, mentorship, and audit and feedback The research approach and content is based on participatory research with HCWs, human-centered design workshops, and stakeholder collaborations [23-29]. This research guided us to include measures of HCW satisfaction and wellbeing. In addition to a HCW experience measure throughout PCPH, the advent of COVID-19 led us to include an assessment of HCW depression. Patients were not directly involved in the design, nor the recruitment of this sub-study assessing the depression levels of HCWs. The

For peer review only - http://bmjopen.bmj.com/site/about/guidelines.xhtml

findings of this study as well as the parent study will be shared with the Zambian Ministry of Health as well as the study facilities.

Ethics

This study was approved as part of the larger PCPH study by the University of Zambia Biomedical Ethics Committee and the University of Alabama at Birmingham Institution Review Board, and the National Health Research Authority in Zambia. We obtained waiver of written informed consent and obtained verbal consent from participants prior to administering the survey.

Results

A total of 713 HCWs from 24 facilities across Lusaka and Chongwe districts in the Lusaka Province were included in the analysis dataset (**Table 1**). The majority (69.6%) of the PHQ-9 survey responses were collected in September 2020 (**Table 1**). The largest proportion of the responses were collected at facilities serving a large client population (41.7%) followed closely by facilities serving s medium-sized client population (37.7%) (**Table 1**). Responses for PHQ-9 questions were largely complete with <1% responses missing.

Table 1: Participant characteristics

Factor	Level	n (%)
N		713
Facility	1	30 (4.2)
	2	30 (4.2)
	3	30 (4.2)
	4	30 (4.2)
	5	30 (4.2)
	6	30 (4.2)
	7	30 (4.2)
	8	30 (4.2)
	9	30 (4.2)
	10	30 (4.2)
	11	30 (4.2)
	12	27 (3.8)
	13	27 (3.8)

For peer review only

For peer review only - http://bmjopen.bmj.com/site/about/guidelines.xhtml

	14	30 (4.2)
	15	30 (4.2)
	16	30 (4.2)
	17	30 (4.2)
	18	30 (4.2)
	19	30 (4.2)
	20	30 (4.2)
	21	30 (4.2)
	22	30 (4.2)
	23	29 (4.1)
	24	30 (4.2)
Clinic Population (category)	Small	147 (20.6)
	Medium	269 (37.7)
	Large	297 (41.7)
Month of Survey	August 2020	205 (28.8)
	September 2020	496 (69.6)
	October 2020	12 (1.7)

Of the 713 responses, 231 (32.4%, 95%CI: 29.0-36.0) reported mild depression (PHQ-9 score 5-9), 81 (11.4% 95%CI: 9.1-13.9) reported moderate depression (PHQ-9 score 10-14). A total of 81 (11.4) respondents had a PHQ-9 score corresponding to moderate depression across all but one facility, 17 (2.4%, 95%CI: 1.4-3.8) respondents had PHQ-9 scores consistent with moderately severe depression across four facilities, and five (0.7%, 95%CI: 0.2-1.6) HCWs had PHQ-9 scores consistent with severe depression across five different facilities (**Fig 1, Fig S1, Table 2**).

Table 2: Proportion of the analysis population by PHQ-9 score category with 95% confidence intervals (N=713)

Variable	*n*	*Proportion (%)*	*95% CI*
≥5 PHQ-9 score	334	46.8	(43.1, 50.6)
Mild	231	32.4	(29.0, 36.0)
Moderate	81	11.4	(9.1, 13.9)
Moderate-severe	17	2.4	(1.4, 3.8)
Severe	5	0.7	(0.2, 1.6)

Note: CI – confidence interval; PHQ-9 – nine-item patient health questionnaire.

For peer review only

For peer review only - http://bmjopen.bmj.com/site/about/guidelines.xhtml

Though we observed facility-level mental health heterogeneity in the proportion of minimal and mild depression scores across clinics, the proportion of scores consistent with moderate depression remain relatively stable across facilities (**Fig 1).**

Figure 1: A) Stacked bar chart of proportion population by PHQ-9 score category by health care facility B) Stacked bar chart of proportion population by PHQ-9 score by facility population size category.

Mixed effects adjusted Poisson regression model did not reveal clinic size or month of survey as a significant predictor of PHQ-9 score ≥5 (**Table 3**).

Table 3: Adjusted Poisson regression results (N=713)

Covariate	*level*	*aPR*	*95% CI*	*p-value*
Clinic Population (category)	small	1.12	(0.95, 1.33)	0.170
	medium	1.00 (ref)	ref	ref
	large	1.04	(0.81, 1.33)	0.763
Month of Survey	August	1.00 (ref)	ref	ref
	September	1.14	(0.94, 1.38)	0.193
	October	0.98	(0.54, 1.79)	0.945

Note: adjusted for survey week and facility

We illustrate significant heterogeneity in the marginal probability of experiencing greater than minimal depression across facilities (**Fig 2**). Notably, the highest marginal probability of HCWs with PHQ-9 score ≥5 was observed in a facility serving as a COVID-19 treatment center.

Figure 2: Marginal probability of health care worker with >minimal depression per PHQ-9 score by clinic.

Note: PHQ-9 – nine-item patient health questionnaire.

Discussion

We found that a large proportion of the HCW population had a PHQ-9 score ≥5 indicating a need for follow-up to assess and improve mental wellbeing 46.8% (95% CI: 43.1, 50.6%). Variation in

For peer review only

For peer review only - http://bmjopen.bmj.com/site/about/guidelines.xhtml

depression outcomes ranged from 82.7% (95% CI: 68.5, 96.9%) at one of the two large COVID-19 treatment facilities to 28.4% (95% CI: 13.0, 43.9%) at a medium-sized health care facility.

Our study shows that HCWs working at the COVID-19 treatment facility had a higher marginal probability of experiencing mild to moderately severe depression. This is consistent with past studies showing that frontline HCW working in clinics managing diseases considered to be highly infectious, such as COVID-19 treatment centers, were more prone to developing depression and other mental disorders than their counterparts in other departments [12]. In addition, results of our study demonstrate that HCWs have experienced symptoms of depression during the onset of the COVID-19 pandemic in Zambia, which is consistent with the findings of similar studies using the PHQ-9 in other parts of the world where a pooled prevalence of mild depression was found to be 36.1% (95% confidence interval [CI], 31.3%-41.0%) [30]. Studies conducted in Ethiopia among different cadres of HCWs show that mental disorders which include depression are prevalent among HCWs during the COVID-19 pandemic, with one study from Ethiopia identifying approximately 48% prevalence of greater than minimal depression, consistent with our Zambian estimates [31, 32]. Similarly, globally studies indicate that there is a risk of HCWs experiencing mental health disorders during the pandemic [4, 30, 31, 33]. The difficult conditions to which HCWs may be exposed including extended working hours, risk of exposure to the disease, increased workload, concerns about transmitting the infection to their family members, reduced social connectedness, and limited resources to care for their patients may amplify poor mental wellbeing [1, 34-36].

Further research is needed to understand heterogeneity in proportions of HCWs with depression. It may be associated variation in HCW access to resources such as, personal protective equipment (PPE), hand hygiene station/station supplies at clinic entrance, and knowledge about the COVID-19 response in Zambia. While the response to the COVID-19 pandemic was standardized, to a certain extent, by guidance from the provincial and zonal levels, the culture and leadership unique to each facility might have played a key role in the health care worker experience, contributing to PHQ-9 score distribution differences across facilities.

For peer review only - http://bmjopen.bmj.com/site/about/guidelines.xhtml

As we continue to recognize the mental health services gap across many populations in resource-limited settings we give evidence here to support prioritization of health care workers, especially during public health shocks/emergencies like that presented by COVID-19.Presenceof depression among HCWs could lead to poor health outcomes for the HCW workforce and have a sort of "knock-on" effect negatively impacting the quality of care provided to patients [37, 38]. Interventions like Friendship Bench piloted in neighboring Zimbabwe designed to encourage positive coping mechanism among HCWs and build a working environment that provides empathy and compassion towards staff may be an efficient option to provide mental wellness support [39, 40]. Furthermore, system-based interventions should also be encouraged such as change in working culture and reduction in possible system contributors to HCW stress that could lead to depression. Increasing mobile technology availability may further allow for the use of mobile health (mHealth) based mental wellness services leveraging the framework presented by Osei for low- and middle-income countries [41, 42]. Low-cost intervention packages used for patients can be adapted for HCWs and integrated into system-based interventions. They include routine screening for early detection, mental wellness education, problem solving and use of anti-depressants, cognitive behavior therapy which can be delivered successfully by trained lay HCWs [43, 44].

Limitations

This study had some potential limitations. Firstly, we did not collect participants demographics such as age, sex, marital status, and sex. As such we were not able to adjust for these co- founders. Accounting for these would help make associations in outcomes of depression. Secondly, we employed convenience sampling in selecting participants. This sampling approach may limit representativeness of study the results to the wider population of HCWs. Finally, without pre-COVID 19 estimates of depression among health care workers in Lusaka, we are not able to show association between mild-severe depression with the pandemic however, we show that during the pandemic that depression was high and attention to this population is justified to ensure a healthy HCW workforce. Additionally, though potentially highest than a non-pandemic baseline, these results may be useful as subsequent measures of depression and mental wellness are collected among HCWs.

For peer review only - http://bmjopen.bmj.com/site/about/guidelines.xhtml

Conclusions

Depression is a common public health problem; our study demonstrates that HCWs in Zambia may suffer from a high prevalence of depressive symptoms that will require additional clinical follow up. Routine mental health wellness is important to better understand the role that the COVID-19 pandemic may have had on depression among HCWS in Zambia. Furthermore, support for HCW mental wellness can serve to accelerate destigmatizing mental health issues and improve the quality of care provided across health care centers in Zambia.

For peer review only

Contributorship

SS: data collection, data Framing, and manuscript writing, JM, KS, and MM: data collection and data curation, LJ and CB: data framing, IS, CBM and EHG: conceptualization, AM: manuscript review/editing, AS: conceptualization and manuscript review/editing LKB: conceptualization, data analysis, data framing, and manuscript writing, JMP: data analysis lead, data framing, and manuscript writing. The corresponding author attests that all listed authors meet authorship criteria and that no others meeting the criteria have been omitted.

Funding

Research reported in this publication was supported by the Bill & Melinda Gates Foundation grant number OPP1166485, the National Institute of Mental Health of the National Institutes of Health under award number F31MH109378 (L.K.B.), and the National Institute of Allergy and Infectious Diseases of the National Institutes of Health under award number K24 AI134413 (E.G.).

Competing of Interests

None

Ethical Approval

This study was approved as part of the larger PCPH study by the University of Zambia Biomedical Ethics Committee and the University of Alabama at Birmingham Institution Review Board, and the National Health Research Authority in Zambia. We obtained waiver of written informed consent and obtained verbal consent from participants prior to administering the survey.

Data Sharing

Extra data can be accessed via the Dryad data repository at http://datadryad.org/ with the doi: https://doi.org/10.25338/B8S646

Acknowledgements

We would like to thank the Zambian Ministry of Health for their leadership to ensure those in care continue to receive life-saving HIV treatment. We would also like to thank the health care

For peer review only

workers for their participation in the PHQ-9 assessment as well as their faithful delivery of care to patients in the face of the many challenges presented by the COVID-19 pandemic. To the clients seen at our study facilities we would like to extend sincere thanks for your cooperation and patience.

List of abbreviations [Optional]

ART: antiretroviral therapy

CI- Confidence Interval

COVID-19: Coronavirus Disease 2019

HCW: Health Care Worker

PCPH: Person- Centered Public Health for HIV Treatment in Zambia

PHQ-9: Patient Health Questionnaire 9

Data Sharing

Data used for this analysis is available through Dryad data repository (doi:10.25338/B8S646)

References

1. Yıldırım M, Çiçek İ, Şanlı ME. Coronavirus stress and COVID-19 burnout among healthcare staffs: The mediating role of optimism and social connectedness. Curr Psychol. 2021;40(11):5763-71. Epub 2021/05/04. doi: 10.1007/s12144-021-01781-w. PubMed PMID: 33935470; PubMed Central PMCID: PMCPMC8064417.

2. Robertson L, Maposa I, Somaroo H, Johnson O. Mental health of healthcare workers during the COVID-19 outbreak: A rapid scoping review to inform provincial guidelines in South Africa. South African Medical Journal. 2020;110(10):1010-9.

3. Gupta S, Sahoo S. Pandemic and mental health of the front-line healthcare workers: a review and implications in the Indian context amidst COVID-19. General Psychiatry. 2020;33(5).

4. Saragih ID, Tonapa SI, Saragih IS, Advani S, Batubara SO, Suarilah I, et al. Global Prevalence of Mental Health Problems among healthcare workers during The Covid-19 Pandemic: A Systematic Review and Meta-Analysis. International Journal of Nursing Studies. 2021:104002.

5. Ayanore MA, Amuna N, Aviisah M, Awolu A, Kipo-Sunyehzi DD, Mogre V, et al. Towards resilient health systems in sub-saharan africa: A systematic review of the english language literature on health workforce, surveillance, and health governance issues for health systems strengthening. Annals of global health. 2019;85(1).

6. Semo B-w, Frissa SM. The mental health impact of the COVID-19 pandemic: implications for sub-Saharan Africa. Psychology Research and Behavior Management. 2020;13:713.

For peer review only

For peer review only - http://bmjopen.bmj.com/site/about/guidelines.xhtml

7. Med S. Serious Health Workforce Shortage in Zambia: Solidar Med; 2020 [updated 22 December 2020; cited 2022 16 January 2022]. Available from: https://www.solidarmed.ch/en/news/shortage-skilled-workers-health-system-zambia.
8. Munakampe MN. Strengthening mental health systems in Zambia. International journal of mental health systems. 2020;14(1):1-9.
9. Deng D, Naslund JA. Psychological impact of COVID-19 Pandemic on frontline health workers in low-and middle-income countries. Harvard public health review (Cambridge, Mass). 2020;28.
10. Moitra M, Rahman M, Collins PY, Gohar F, Weaver M, Kinuthia J, et al. Mental health consequences for healthcare workers during the COVID-19 pandemic: a scoping review to draw lessons for LMICs. Frontiers in psychiatry. 2021;12:22.
11. Ceri V, Cicek I. Psychological Well-Being, Depression and Stress During COVID-19 Pandemic in Turkey: A Comparative Study of Healthcare Professionals and Non-Healthcare Professionals. Psychology, health & medicine. 2021;26(1):85-97. Epub 2020/12/16. doi: 10.1080/13548506.2020.1859566. PubMed PMID: 33320723.
12. Shah J, Monroe-Wise A, Talib Z, Nabiswa A, Said M, Abeid A, et al. Mental health disorders among healthcare workers during the COVID-19 pandemic: a cross-sectional survey from three major hospitals in Kenya. BMJ open. 2021;11(6):e050316.
13. Chersich MF, Gray G, Fairlie L, Eichbaum Q, Mayhew S, Allwood B, et al. COVID-19 in Africa: care and protection for frontline healthcare workers. Global Health. 2020;16(1):46. Epub 2020/05/18. doi: 10.1186/s12992-020-00574-3. PubMed PMID: 32414379; PubMed Central PMCID: PMCPMC7227172.
14. Yitayih Y, Mekonen S, Zeynudin A, Mengistie E, Ambelu A. Mental health of healthcare professionals during the early stage of the COVID-19 pandemic in Ethiopia. BJPsych Open. 2020;7(1):e1. Epub 2020/12/02. doi: 10.1192/bjo.2020.130. PubMed PMID: 33256883; PubMed Central PMCID: PMCPMC7844150.
15. Jemal K, Deriba BS, Geleta TA, Tesema M, Awol M, Mengistu E, et al. Self-Reported Symptoms of Depression, Anxiety, and Stress Among Healthcare Workers in Ethiopia During the COVID-19 Pandemic: A Cross-Sectional Study. Neuropsychiatr Dis Treat. 2021;17:1363-73. Epub 2021/05/15. doi: 10.2147/NDT.S306240. PubMed PMID: 33986596; PubMed Central PMCID: PMCPMC8110274.
16. Asnakew S, Amha H, Kassew T. Mental Health Adverse Effects of COVID-19 Pandemic on Health Care Workers in North West Ethiopia: A Multicenter Cross-Sectional Study. Neuropsychiatr Dis Treat. 2021;17:1375-84. Epub 2021/05/18. doi: 10.2147/NDT.S306300. PubMed PMID: 33994787; PubMed Central PMCID: PMCPMC8113010.
17. Sahebi A, Nejati-Zarnaqi B, Moayedi S, Yousefi K, Torres M, Golitaleb M. The prevalence of anxiety and depression among healthcare workers during the COVID-19 pandemic: An umbrella review of meta-analyses. Prog Neuropsychopharmacol Biol Psychiatry. 2021;107:110247. Epub 2021/01/22. doi: 10.1016/j.pnpbp.2021.110247. PubMed PMID: 33476692; PubMed Central PMCID: PMCPMC7817526.
18. Elbay RY, Kurtulmuş A, Arpacıoğlu S, Karadere E. Depression, anxiety, stress levels of physicians and associated factors in Covid-19 pandemics. Psychiatry research. 2020;290:113130.
19. Kroenke K, Spitzer RL, Williams JB. The PHQ-9: validity of a brief depression severity measure. Journal of general internal medicine. 2001;16(9):606-13. doi: 10.1046/j.1525-1497.2001.016009606.x. PubMed PMID: 11556941.

For peer review only

For peer review only - http://bmjopen.bmj.com/site/about/guidelines.xhtml

20. Kroenke K, Spitzer RL, Williams JB. The PHQ-9: validity of a brief depression severity measure. Journal of general internal medicine. 2001;16(9):606-13.
21. Kroenke K, Spitzer RL, Williams JB, Löwe B. The patient health questionnaire somatic, anxiety, and depressive symptom scales: a systematic review. General hospital psychiatry. 2010;32(4):345-59.
22. Sawka KS, Mbewe C, Josephat D, Ngulube E. The Senga of Zambia: Linguistic Survey Report with Recommendations for Literature Development Strategy. SIL International: 2021.
23. Beres LK, Simbeza S, Holmes CB, Mwamba C, Mukamba N, Sharma A, et al. Human-centered design lessons for implementation science: improving the implementation of a patient-centered care intervention. Journal of acquired immune deficiency syndromes (1999). 2019;82(3):S230.
24. Mody A, Tram KH, Glidden DV, Eshun-Wilson I, Sikombe K, Mehrotra M, et al. Novel Longitudinal Methods for Assessing Retention in Care: a Synthetic Review. Current HIV/AIDS Reports. 2021;18(4):299-308.
25. Sikombe K, Mody A, Kadota J, Pry JJ, Simbeza S, Eshun-Wilson I, et al. Understanding patient transfers across multiple clinics in Zambia among HIV infected adults. PloS one. 2020;15(11):e0241477.
26. Holmes CB, Sanne I. Changing models of care to improve progression through the HIV treatment cascade in different populations. Current Opinion in HIV and AIDS. 2015;10(6):447-50.
27. Holmes CB, Sikazwe I, Sikombe K, Eshun-Wilson I, Czaicki N, Beres LK, et al. Estimated mortality on HIV treatment among active patients and patients lost to follow-up in 4 provinces of Zambia: Findings from a multistage sampling-based survey. PLoS Med. 2018;15(1):e1002489. Epub 2018/01/13. doi: 10.1371/journal.pmed.1002489. PubMed PMID: 29329301; PubMed Central PMCID: PMCPMC5766235.
28. Roy M, Czaicki N, Holmes C, Chavan S, Tsitsi A, Odeny T, et al. Understanding Sustained Retention in HIV/AIDS Care and Treatment: a Synthetic Review. Current HIV/AIDS Reports. 2016:1-9.
29. Mwamba C, Sharma A, Mukamba N, Beres L, Geng E, Holmes CB, et al. 'They care rudely!': resourcing and relational health system factors that influence retention in care for people living with HIV in Zambia. BMJ Glob Health. 2018;3(5):e001007. Epub 2018/11/30. doi: 10.1136/bmjgh-2018-001007. PubMed PMID: 30483408; PubMed Central PMCID: PMCPMC6231098.
30. Li Y, Scherer N, Felix L, Kuper H. Prevalence of depression, anxiety and post-traumatic stress disorder in health care workers during the COVID-19 pandemic: A systematic review and meta-analysis. PloS one. 2021;16(3):e0246454.
31. Mulatu HA, Tesfaye M, Woldeyes E, Bayisa T, Fisseha H, Kassu RA. The prevalence of common mental disorders among healthcare professionals during the COVID-19 pandemic at a tertiary Hospital in Addis Ababa, Ethiopia. Journal of Affective Disorders Reports. 2021;6:100246. doi: https://doi.org/10.1016/j.jadr.2021.100246.
32. Yadeta TA, Dessie Y, Balis B. Magnitude and Predictors of Health Care Workers Depression During the COVID-19 Pandemic: Health Facility-Based Study in Eastern Ethiopia. Frontiers in Psychiatry. 2021;12. doi: 10.3389/fpsyt.2021.654430.
33. Fond G, Fernandes S, Lucas G, Greenberg N, Boyer L. Depression in healthcare workers: Results from the nationwide AMADEUS survey. International journal of nursing studies.

For peer review only - http://bmjopen.bmj.com/site/about/guidelines.xhtml

2022;135:104328. Epub 2022/08/12. doi: 10.1016/j.ijnurstu.2022.104328. PubMed PMID: 35952535; PubMed Central PMCID: PMCPMC9359895.
34. Mokhtari R, Moayedi S, Golitaleb M. COVID-19 pandemic and health anxiety among nurses of intensive care units. International journal of mental health nursing. 2020;29(6):1275.
35. Pfefferbaum B, North CS. Mental health and the Covid-19 pandemic. New England Journal of Medicine. 2020;383(6):510-2.
36. Yıldırım M, Çiçek İ. Optimism and pessimism mediate the association between parental coronavirus anxiety and depression among healthcare professionals in the era of COVID-19. Psychology, health & medicine. 2022;27(9):1898-906. Epub 2021/08/13. doi: 10.1080/13548506.2021.1966702. PubMed PMID: 34382894.
37. Saasa Y. An Assessment on the Role of Human Rights and Governance in Health Service Delivery 2019.
38. Kola L, Kohrt BA, Hanlon C, Naslund JA, Sikander S, Balaji M, et al. COVID-19 mental health impact and responses in low-income and middle-income countries: reimagining global mental health. The Lancet Psychiatry. 2021;8(6):535-50. doi: https://doi.org/10.1016/S2215-0366(21)00025-0.
39. Abraham A, Chaabna K, Doraiswamy S, Bhagat S, Sheikh J, Mamtani R, et al. Depression among healthcare workers in the Eastern Mediterranean Region: a systematic review and meta-analysis. Human resources for health. 2021;19(1):1-18.
40. Wallén A, Eberhard S, Landgren K. The Experiences of Counsellors Offering Problem-Solving Therapy for Common Mental Health Issues at the Youth Friendship Bench in Zimbabwe. Issues in Mental Health Nursing. 2021;42(9):808-17. doi: 10.1080/01612840.2021.1879977.
41. Osei E, Mashamba-Thompson TP. Mobile health applications for disease screening and treatment support in low-and middle-income countries: A narrative review. Heliyon. 2021;7(3):e06639. Epub 2021/04/20. doi: 10.1016/j.heliyon.2021.e06639. PubMed PMID: 33869857; PubMed Central PMCID: PMCPMC8035664.
42. Mutimushi PM, Mupenda BM, Manda PM, Nankonde N, Banda B, Mung'omba K, et al. ZICTA 2019 Annual Report. https://www.zicta.zm/storage/posts/attachments/oiBT4oMnpJoN3dgHKpvByoDmNXq5ZDYtp3sxVYM3.pdf: Zambia Information and Communications Technology Authority (ZICTA), 2019.
43. Chibanda D, Mesu P, Kajawu L, Cowan F, Araya R, Abas MA. Problem-solving therapy for depression and common mental disorders in Zimbabwe: piloting a task-shifting primary mental health care intervention in a population with a high prevalence of people living with HIV. BMC public health. 2011;11(1):1-10.
44. Patel V, Simon G, Chowdhary N, Kaaya S, Araya R. Packages of care for depression in low-and middle-income countries. PLoS medicine. 2009;6(10):e1000159.

Supplement:

Figure S1: Histogram of total PHQ-9 scores among study participants

For peer review only - http://bmjopen.bmj.com/site/about/guidelines.xhtml

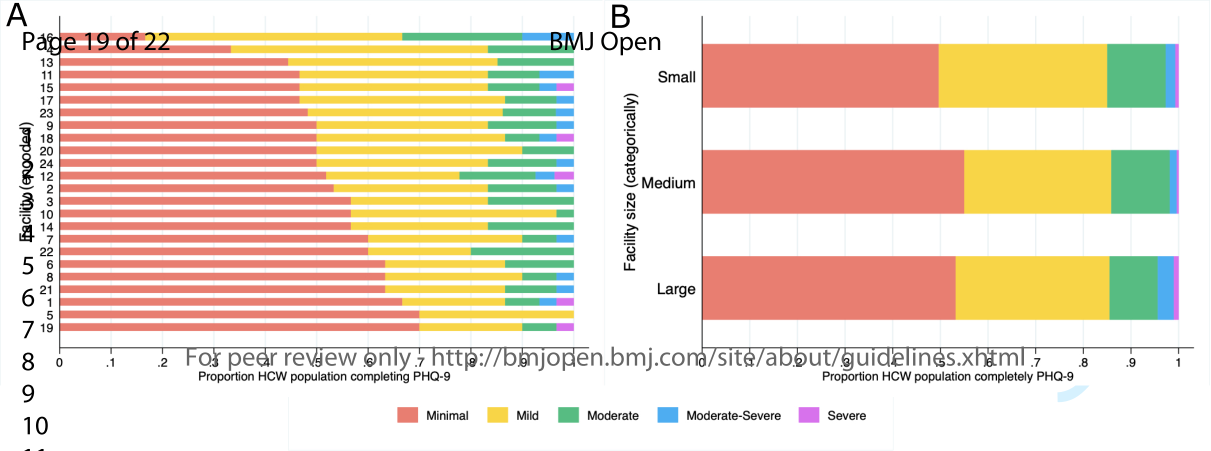

For peer review only - http://bmjopen.bmj.com/site/about/guidelines.xhtml

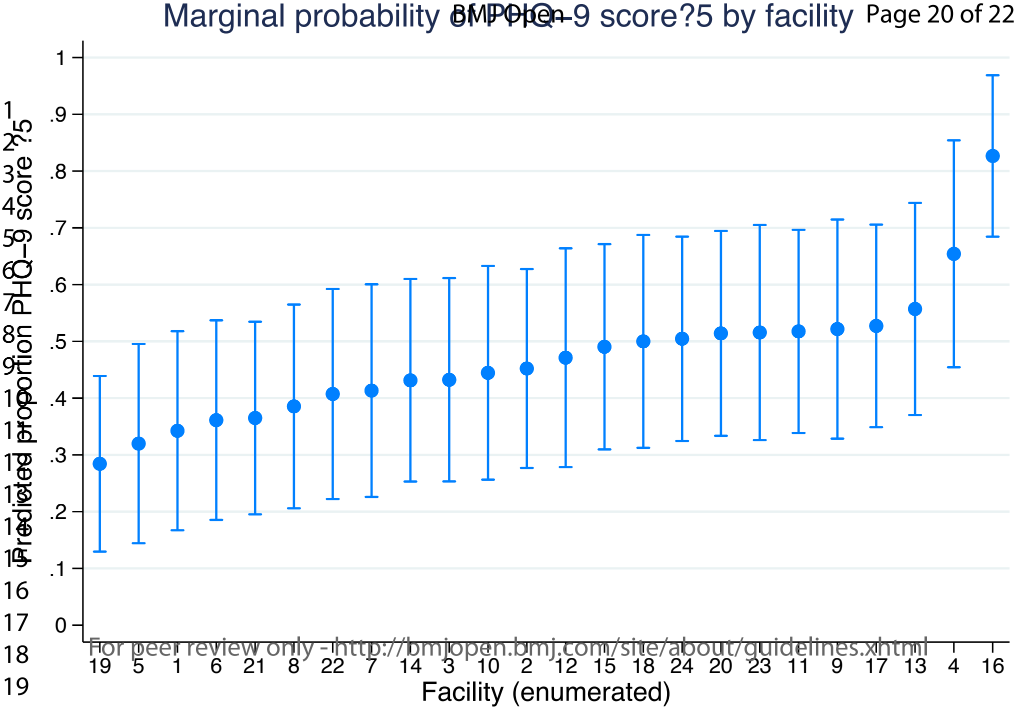

For peer review only - http://bmjopen.bmj.com/site/about/guidelines.xhtml

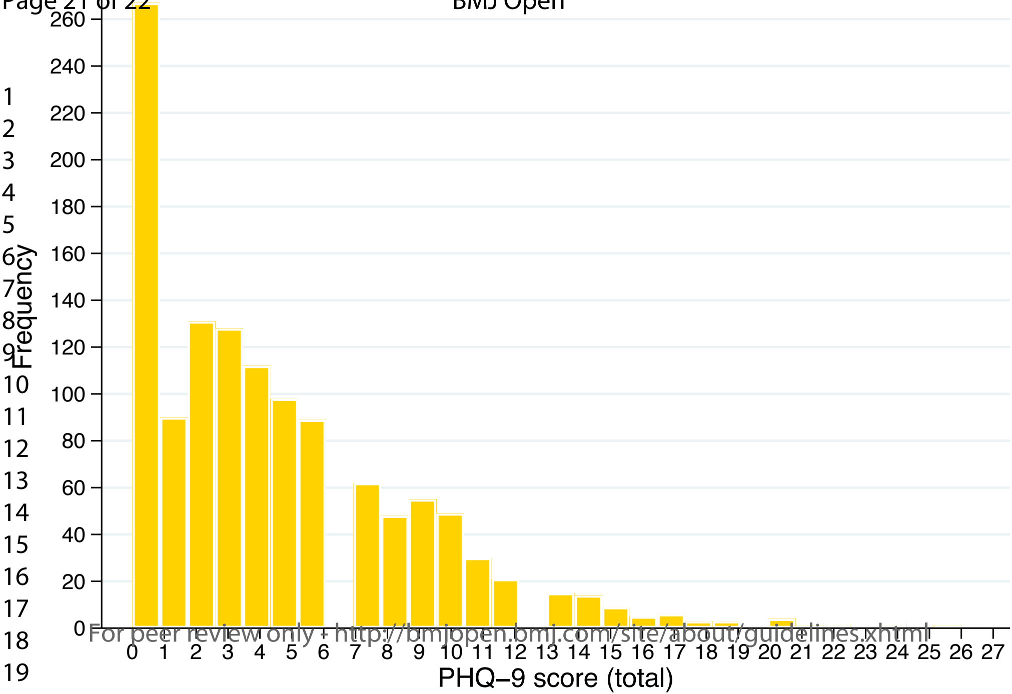

For peer review only - http://bmjopen.bmj.com/site/about/guidelines.xhtml

STROBE Statement—Checklist of items that should be included in reports of ***cohort studies***

	Item No	Recommendation	Page No
**Title and abstract**	1	(*a*) Indicate the study's design with a commonly used term in the title or the abstract	2
		(*b*) Provide in the abstract an informative and balanced summary of what was done and what was found	2
**Introduction**			
Background/rationale	2	Explain the scientific background and rationale for the investigation being reported	4
Objectives	3	State specific objectives, including any prespecified hypotheses	4, 5
**Methods**			
Study design	4	Present key elements of study design early in the paper	5
Setting	5	Describe the setting, locations, and relevant dates, including periods of recruitment, exposure, follow-up, and data collection	5
Participants	6	(*a*) Give the eligibility criteria, and the sources and methods of selection of participants. Describe methods of follow-up	5, 6
		(*b*) For matched studies, give matching criteria and number of exposed and unexposed	NA
Variables	7	Clearly define all outcomes, exposures, predictors, potential confounders, and effect modifiers. Give diagnostic criteria, if applicable	5, 6
Data sources/ measurement	8*	For each variable of interest, give sources of data and details of methods of assessment (measurement). Describe comparability of assessment methods if there is more than one group	5, 6
Bias	9	Describe any efforts to address potential sources of bias	5, 6
Study size	10	Explain how the study size was arrived at	5
Quantitative variables	11	Explain how quantitative variables were handled in the analyses. If applicable, describe which groupings were chosen and why	5, 6
Statistical methods	12	(*a*) Describe all statistical methods, including those used to control for confounding	6
		(*b*) Describe any methods used to examine subgroups and interactions	6
		(*c*) Explain how missing data were addressed	6
		(*d*) If applicable, explain how loss to follow-up was addressed	NA
		(*e*) Describe any sensitivity analyses	6
**Results**			
Participants	13*	(a) Report numbers of individuals at each stage of study—eg numbers potentially eligible, examined for eligibility, confirmed eligible, included in the study, completing follow-up, and analysed	7, 8
		(b) Give reasons for non-participation at each stage	NA
		(c) Consider use of a flow diagram	NA
Descriptive data	14*	(a) Give characteristics of study participants (eg demographic, clinical, social) and information on exposures and potential confounders	7, 8
		(b) Indicate number of participants with missing data for each variable of interest	7, 8
		(c) Summarise follow-up time (eg, average and total amount)	NA
Outcome data	15*	Report numbers of outcome events or summary measures over time	7, 8

For peer review only - http://bmjopen.bmj.com/site/about/guidelines.xhtml

Main results	16	(*a*) Give unadjusted estimates and, if applicable, confounder-adjusted estimates and their precision (eg, 95% confidence interval). Make clear which confounders were adjusted for and why they were included	7, 8
		(*b*) Report category boundaries when continuous variables were categorized	7, 8
		(*c*) If relevant, consider translating estimates of relative risk into absolute risk for a meaningful time period	NA
Other analyses	17	Report other analyses done—eg analyses of subgroups and interactions, and sensitivity analyses	9
**Discussion**			
Key results	18	Summarise key results with reference to study objectives	10
Limitations	19	Discuss limitations of the study, taking into account sources of potential bias or imprecision. Discuss both direction and magnitude of any potential bias	11, 12
Interpretation	20	Give a cautious overall interpretation of results considering objectives, limitations, multiplicity of analyses, results from similar studies, and other relevant evidence	9, 10
Generalisability	21	Discuss the generalisability (external validity) of the study results	9, 10
**Other information**			
Funding	22	Give the source of funding and the role of the funders for the present study and, if applicable, for the original study on which the present article is based	13, 14

*Give information separately for exposed and unexposed groups.

Note: An Explanation and Elaboration article discusses each checklist item and gives methodological background and published examples of transparent reporting. The STROBE checklist is best used in conjunction with this article (freely available on the Web sites of PLoS Medicine at http://www.plosmedicine.org/, Annals of Internal Medicine at http://www.annals.org/, and Epidemiology at http://www.epidem.com/). Information on the STROBE Initiative is available at http://www.strobe-statement.org.

For peer review only
